# Phenotypic switch of vascular smooth muscle cells in COVID‐19: Role of cholesterol, calcium, and phosphate

**DOI:** 10.1002/jcp.31424

**Published:** 2024-08-26

**Authors:** Laura Ghanem, Dina Essayli, Jana Kotaich, Mohammad Al Zein, Amirhossein Sahebkar, Ali H. Eid

**Affiliations:** ^1^ Faculty of Medical Sciences Lebanese University Hadath Lebanon; ^2^ MEDICA Research Investigation Beirut Lebanon; ^3^ Center for Global Health Research, Saveetha Medical College and Hospitals, Saveetha Institute of Medical and Technical Sciences Saveetha University Chennai India; ^4^ Biotechnology Research Center, Pharmaceutical Technology Institute Mashhad University of Medical Sciences Mashhad Iran; ^5^ Applied Biomedical Research Center Mashhad University of Medical Sciences Mashhad Iran; ^6^ Department of Basic Medical Sciences, College of Medicine, QU Health Qatar University Doha Qatar

**Keywords:** atherosclerosis, calcification, cardiovascular disease, differentiation, SARS‐CoV‐2

## Abstract

Although the novel coronavirus disease 2019 (COVID‐19), caused by the severe acute respiratory syndrome coronavirus 2 (SARS‐CoV‐2), primarily manifests as severe respiratory distress, its impact on the cardiovascular system is also notable. Studies reveal that COVID‐19 patients often suffer from certain vascular diseases, partly attributed to increased proliferation or altered phenotype of vascular smooth muscle cells (VSMCs). Although the association between COVID‐19 and VSMCs is recognized, the precise mechanism underlying SARS‐CoV‐2's influence on VSMC phenotype remains largely under‐reviewed. In this context, while there is a consistent body of literature dissecting the effect of COVID‐19 on the cardiovascular system, few reports delve into the potential role of VSMC switching in the pathophysiology associated with COVID‐19 and the molecular mechanisms involved therein. This review dissects and critiques the link between COVID‐19 and VSMCs, with particular attention to pathways involving cholesterol, calcium, and phosphate. These pathways underpin the interaction between the virus and VSMCs. Such interaction promotes VSMC proliferation, and eventually potentiates vascular calcification as well as worsens prognosis in patients with COVID‐19.

**Abbreviations**
25HC: 25‐hydroxycholesterol
27OHC: 27‐hydroxycholesterol
ACAT: acyl‐CoA cholesterol acyltransferase
ACE2: angiotensin‐converting enzyme 2
ACTA2: smooth muscle aortic alpha‐actin
ADAM17: disintegrin and metalloproteinase domain‐containing protein 17
ALP: alkaline phosphatase
AMC: arterial medial calcification
ApoB: apolipoprotein B
ATP: adenosine 5′‐triphosphate
Ca: calcium
CAC: coronary artery calcification
cAMP: cyclic adenosine monophosphate
CH25H: cholesterol 25‐hydroxylase
ChemR23: chemerin receptor 23
CKD: chronic kidney disease
COL1: type 1 collagen
COVID‐19: coronavirus disease of 2019
CREB: cAMP response element‐binding protein
CVDs: cardiovascular diseases
ECM: extracellular matrix
ER: endoplasmic reticulum
Fe^2+^
: ferrous ions
FN: fibronectin
GPI: glycosylphosphatidylinositol
HcoV‐OC43: human coronavirus organ culture 43
HDL: high‐density lipoprotein
HF: heart failure
HIF‐1: hypoxia‐inducible factor‐1
HiPO^4−^
: inorganic phosphate
HSPG: heparan sulfate proteoglycan
IL: interleukin
ISG: interferon‐stimulated gene
LDL‐c: low‐density lipoprotein cholesterol
LDL‐P: low‐density lipoprotein particle
LOE: lindera obtusiloba
LXR: liver X receptor
MGP: matrix Gla protein
mtROS: mitochondrial reactive oxygen species
MYH11: myosin heavy chain
MβCD: methyl‐β‐cyclodextrin
NF‐B: nuclear factor kappa B
O2: dioxygen
OPN: osteopontin
PDGF‐BB: platelet‐derived growth factor BB
PKA: protein kinase A
PMVs: platelet‐derived microvesicles
Ppi: inhibitor pyrophosphate
RAAS: renin‐angiotensin‐aldosterone
RBD: receptor binding domain
Runx2: runt‐related transcription factor 2
SARS‐CoV‐2: severe acute respiratory syndrome coronavirus 2
SM22α: smooth muscle 22 alpha
SMC: smooth muscle cell
SOD2: superoxide dismutase 2
Sox9: SRY‐box transcription factor 9
SREBP2: sterol‐regulatory element binding proteins‐2
T3: triiodothyronine
TGF‐β1: transforming growth factor beta 1
TLR4: toll‐like receptor 4
TNF‐α: tumor necrosis factor‐alpha
TRPV4: transient receptor potential vanilloid 4
UBIAD1: UbiA prenyltransferase domain containing 1
UTP: uridine‐5′‐triphosphate
VEGF: vascular endothelial growth factor
VSMCs: vascular smooth muscle cells

## 1 INTRODUCTION

Coronavirus disease 2019 (COVID‐19) is a contagious disease caused by the severe acute respiratory syndrome coronavirus 2 (SARS‐CoV‐2), a Beta‐coronavirus genus in the Coronaviridae family (Martinez‐Salazar et al., 2022; SeyedAlinaghi et al., 2021). SARS‐CoV‐2 is an enveloped, positive‐sense, single‐stranded ribonucleic acid (RNA) virus (Harrison et al., 2020). Two‐thirds of its genome encodes 16 nonstructural proteins (Harrison et al., 2020). Four structural proteins, namely the spike (S), envelope (E), membrane (M), and nucleocapsid (N), are encoded by the remaining one‐third of the genome (Harrison et al., 2020). The spike S protein plays an essential role in mediating the entry of SARS‐CoV‐2 into the target cells (Harrison et al., 2020). In addition, another component that allows the virus to “enter” target cells is the angiotensin‐converting enzyme 2 (ACE2) receptor (Salabei et al., 2022). This receptor is more ubiquitously expressed on myocytes, fibroblasts, endothelial cells, and smooth muscle cells (SMCs) (Salabei et al., 2022).

Respiratory involvement remains the primary manifestation of COVID‐19. According to a systematic review and meta‐analysis, more than half of patients with COVID‐19 present with cough (da Rosa Mesquita et al., 2021). However, COVID‐19 has also been shown to promote systemic symptoms. In essence, robust evidence suggests that significant portion of SARS‐CoV‐2 morbidity and mortality is attributed to cardiovascular complications, such as acute coronary syndrome, myocarditis, arrhythmia, and venous thromboembolism (Clerkin et al., 2020). A meta‐analysis reported that the prevalence of cardiac injury in hospitalized patients with COVID‐19 is 22%, and this proportion increases with disease severity, suggesting that cardiac injury is associated with worse prognosis and higher mortality (Fu et al., 2021). Furthermore, CVD is the most common comorbidity in patients with COVID‐19, and COVID‐19 patients with underlying cardiovascular disease demonstrate a worse prognosis (Li, Dong, et al., 2020). Among the mechanisms proposed to explain the bidirectional interaction between COVID‐19 and the cardiovascular system, the ACE2 receptor appears to play a key role, as evidenced by both animal and human studies (Chilazi et al., 2021). The presence of ACE2 on the surface of endothelial cells and vascular smooth muscle cells (VSMCs) mediates cardiovascular injury and contributes to increased mortality (Liu et al., 2020).

VSMCs play integral roles in regulating vasotone and blood flow (Deng et al., 2021; Zhang et al., 2016). In healthy vasculature, VSMCs perform many of the physical homeostatic functions of arteries and arterioles, as well as the production and remodeling of the extracellular matrix (ECM) (Deng et al., 2021; Zhang et al., 2016). Under certain environmental factors, these highly specialized quiescent cells may undergo certain morphological, followed by functional, alterations that may lead to VSMC‐driven vascular diseases (Deng et al., 2021; Zhang et al., 2016). This plasticity of VSMCs is most evident when they respond to various stimuli by switching from a differentiated to a dedifferentiated phenotype (Frismantiene et al., 2018; Zhang et al., 2016). In addition, when blood vessels are damaged or stimulated by growth factors, VSMCs can respond by increased proliferation, migration, and synthesis of extracellular components, a phenomenon referred to as phenotypic switching (Frismantiene et al., 2018; Zhang et al., 2016). Many critical regulators can be implicated in the pathogenesis of this phenotypic switching, such as ions and molecules, including cholesterol, calcium, and phosphate.

The role of cholesterol, calcium, and phosphate in VSMC phenotypic switch has been extensively studied (Tang et al., 2022; Vengrenyuk et al., 2015). In patients with atherosclerosis, VSMC dedifferentiation plays a pivotal role in plaque formation. Indeed, it was shown that in atherosclerotic patients, approximately 40% of cells identified as macrophages were derived from VSMCs (Vengrenyuk et al., 2015). Interestingly, one molecule that plays a role in VSMCs transition from a contractile phenotype to a macrophage‐like one is cholesterol (Vengrenyuk et al., 2015). Moreover, contractile VSMCs may exhibit resistance to vascular calcification, a process wherein calcium and phosphate are deposited in the form of hydroxyapatites (Furmanik et al., 2020). Intriguingly, phenotypic remodeling of VSMCs promotes vascular calcification (Furmanik et al., 2020), while calcification upregulates transcription factors that mediate differentiation of VSMCs to an osteoblastic phenotype (Bundy et al., 2021). Therefore, an imbalance of calcium and phosphate may also play an important role in the VSMC phenotype switching, and vice versa (Bundy et al., 2021; Furmanik et al., 2020).

Although the relationship between COVID‐19 and arterial muscle cells has been suggested in recent studies, the exact mechanism of the interplay between SARS‐CoV‐2 and VSMC phenotypic switching remains largely unknown (Kar, 2022; Martínez‐Salazar et al., 2022; Naeem et al., 2023). Thus, this review was undertaken to determine the molecular mechanism implicated in this switch while highlighting the active role of calcium, phosphate, and cholesterol in the pathophysiology of vascular outcomes and disease progression in COVID‐19 patients. Moreover, pinpointing the dysregulation of VSMC modulation in patients with comorbidities may explain why COVID‐19 is more serious in these individuals.

## 2 COVID‐19 AND THE CARDIOVASCULAR SYSTEM

As mentioned earlier, cardiovascular involvement is a common complication of COVID‐19, and this is attributed to the powerful expression of ACE2 receptor on the surface of epithelial and vascular smooth muscle cells (Chatzis et al., 2022; Salabei et al., 2022; Wehbe, Hammoud, et al., 2021). The interaction between SARS‐CoV‐2 and ACE2 disrupts signal transduction pathways and cellular hemostasis, resulting in myocardial and vascular injury (Soumya et al., 2021). Furthermore, other mechanisms such as hypoxia and systemic inflammation put an additional burden on the heart and vessels, leading to a worse prognosis (Nishiga et al., 2020). Acute cardiac injury, defined by the elevation of serum cardiac biomarker levels >99th percentile of upper reference limit, is a common extrapulmonary manifestation of COVID‐19 (Tajbakhsh et al., 2021). About 21% of patients hospitalized for COVID‐19 demonstrate cardiac injury, and this proportion rises to almost 100% in critically ill patients (Li, Yang, et al., 2020). The pathophysiological mechanisms underlying myocardial injury in COVID‐19 patients include direct viral damage, endothelial dysfunction, hypoxia, and systemic inflammation (Helms et al., 2022). Clinical evidence has also demonstrated that SARS‐CoV‐2 can trigger acute coronary syndrome, and this has been attributed to endothelial injury, thrombus formation, and plaque rapture (Schiavone et al., 2020). In addition, patients with COVID‐19 are at increased risk of arrhythmias and sudden cardiac arrest. According to a systematic review, the incidence of arrhythmias in patients with COVID‐19 is up to 18%, and this can be attributed to myocardial injury, electrolyte imbalances, fever, and sepsis (Hessami et al., 2021). Acute or decompensated heart failure, as evidenced by the development of cardiogenic pulmonary edema, has been reported in 6.5% of COVID‐19 patients (Harrison et al., 2021). The development of systemic symptoms, such as fever, tachycardia, and hypoxia, in patients with underlying comorbidities might result in the decompensation of cardiac function. Furthermore, myocardial injury and acute coronary syndrome triggered by COVID‐19 can also result in cardiac dysfunction (Mehra & Ruschitzka, 2020). Clinical observations showed that COVID‐19 patients are also at increased risk of venous thromboembolism. Studies from China demonstrate that high levels of D‐dimer (≥0.5 mg/l) were present in 46% of patients (Guan et al., 2020). In another study, elevated D‐dimer was associated with greater odds of death (Zhou et al., 2020). It is suggested that the systemic inflammatory response and endothelial dysfunction promote a condition of hypercoaculable state in COVID‐19 patients (Bikdeli et al., 2020).

Several studies highlighted that the course of COVID‐19 is not only dependent on the effect of SARS‐CoV‐2 infection on the host cells, but also on the downstream signaling pathways initiated by the binding of coronavirus' spike protein to its receptor on the endothelial cells and VSMCs (Parums, 2022; Suzuki et al., 2021). The endothelial cells, which constitute the innermost layer of the blood vessels, play a pivotal role in maintaining tissue homeostasis, through the regulation of systemic blood flow, immune system, coagulability state, and tissue perfusion, in accordance with other cells, such as pericytes and VSMCs (Pelisek et al., 2022; Soumya et al., 2021; Xu et al., 2023). However, disruptions in cellular homeostasis in the settings of COVID‐19, such as the upregulation of reactive oxygen species production and subsequent imbalance in redox status, promote endothelial dysfunction and organ injury (Soumya et al., 2021). Furthermore, accumulating evidence suggests that SARS‐CoV‐2 infection causes various instances of VSMC dysfunction, including phenotypic switch, proliferation, and hypertrophy. These modifications are deemed to increase contractility and induce vascular remodeling. Essentially, a study highlighted that SARS‐CoV‐2 infection promotes vascular dysfunction characterized by enhanced vasoconstriction and impaired vasorelaxation by acting on the RhoA/Rho‐kinase signaling pathway (Sykes et al., 2023). Furthermore, some studies focused on the involvement of SARS‐CoV‐2 in enhancing the systemic inflammation mediated by the NLRP3 inflammasome in VSMCs via the SCAP‐SREBP signaling pathway; which can put into relief the interconnectedness between COVID‐19 and VSMCs (Liu et al., 2024a, 2024b). Another study elucidated the mechanisms underlying the increased cardiovascular risk after coronavirus infection by focusing on the IL18/IL18R1/HIF‐1 signaling pathway (Zhang et al., 2021). Essentially, IL18 induction during SARS‐CoV‐2 infection results in the abnormal activation of HIF‐1 signaling pathway, thereby promoting the synthetic phenotype of VSMC. Interestingly, HIF‐1 overexpression has been demonstrated a key mechanism in the pathogensis of vascular disease, such as atherosclerosis and aneurysm formation (Gao et al., 2012).

## 3 COVID‐19 AND VSMC PHENOTYPIC SWITCH: A FOCUS ON CHOLESTEROL, CALCIUM, AND PHOSPHATE

### 3.1 Role of cholesterol and calcium in SARS‐CoV‐2 entry and replication

**Figure 1:**
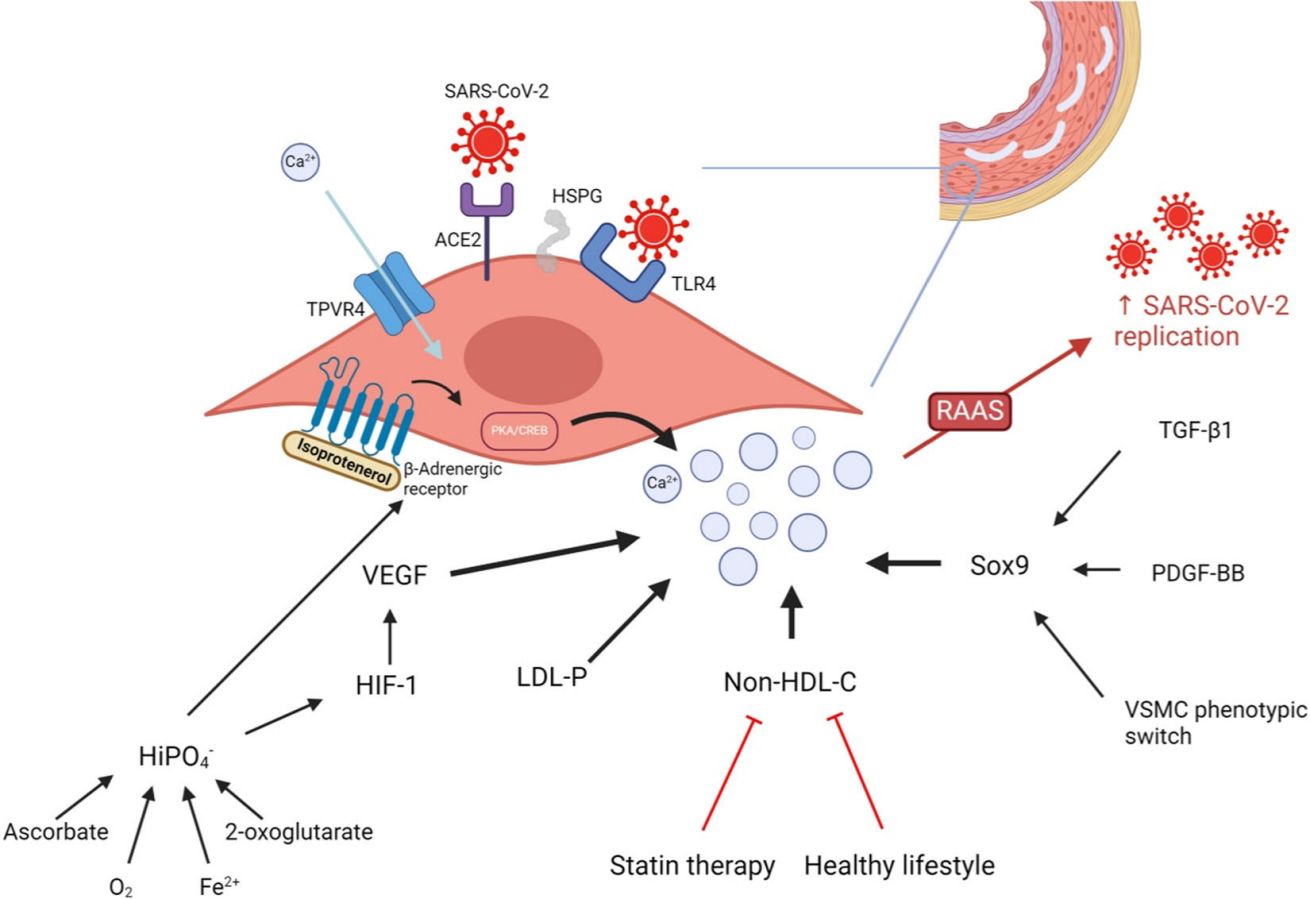
Potential mechanisms promoting vascular smooth muscle cells (VSMC) calcification and its phenotype switch, resulting in increased severe acute respiratory syndrome coronavirus 2 (SARS‐CoV‐2) replication and entry. Calcification plays an important role in the VSMCs phenotypic switch. On one hand, an increased Ca^2+^ flow can be mediated through the transient receptor potential vanilloid 4 (TRPV4) on the VSMCs surface, resulting in their dedifferentiation. On the other hand, phenotypic switch can itself promote Ca^2+^ deposition. This calcification further enhances the SARS‐CoV‐2 replication via the renin‐angiotensin‐aldosterone (RAAS) activation. Moreover, lipid molecules can have a stimulatory effect on calcium, as well as on the coronavirus entry through toll‐like receptor 4 (TLR4) and angiotensin‐converting enzyme 2 (ACE2) receptor enhanced by heparan sulfate proteoglycan (HSPG). In fact, other mechanisms can also stimulate calcium deposits. For instance, ascorbate, O_2_, Fe^2+^, and 2‐oxyglutarate result in hyperphosphatemia, which activates the hypoxia‐inducible factor‐1 (HIF‐1), responsible of stimulating the vascular endothelial growth factor (VEGF), an important factor for calcification. Moreover, high HiPO^4−^ levels heighten beta‐adrenergic receptor activity, thus stimulating the protein kinase A (PKA)/cAMP response element‐binding protein (CREB) pathway and increasing Ca^2+^ levels. SRY‐box transcription factor 9 (Sox9) expression is also involved in Ca^2+^ deposition. Created with BioRender.com. Ca, calcium; Fe^2+^, ferrous ions; HDL, high‐density lipoprotein; HiPO^4−^, inorganic phosphate; LDL‐P: low‐density lipoprotein particle; O_2_, dioxygen; PDGF‐BB, platelet‐derived growth factor BB; TGF‐β1, transforming growth factor beta 1.

One of the factors that play a role in SARS‐CoV‐2 infection is lipid metabolism, particularly the one involving cholesterol. Indeed, it was demonstrated that the coronavirus entrance into the cell involves plasma fusion and endocytosis, a mechanism that primarily involves the lipid raft microdomains characterized by the presence of cholesterol, glycosphingolipids, and glycosylphosphatidylinositol (GPI)‐anchored proteins (Casari et al., 2021; Kočar et al., 2021). These domains are considered the docking site of SARS‐CoV‐2 to pass through the cell membrane and release their genome (Casari et al., 2021; Kočar et al., 2021). In effect, it was shown that the cholesterol‐rich lipid rafts are highly concentrated in receptors and co‐receptors (Casari et al., 2021; Clausen et al., 2020; Li, Zhu, et al., 2021; Palacios‐Rápalo et al., 2021). These receptors could have synergistic effects with the SARS‐CoV‐2 surface proteins and modulate the virus entry through the cell membrane (Figure 1) (Casari et al., 2021; Clausen et al., 2020; Li, Zhu, et al., 2021; Palacios‐Rápalo et al., 2021). For example, one of these receptors, particularly the ACE2 receptor, binds to the S protein‐receptor binding domain (RBD) of SARS‐CoV‐2 and facilitates its entry (Casari et al., 2021; Clausen et al., 2020; Palacios‐Rápalo et al., 2021). Viral entry through this receptor also necessitates the interaction between the coronavirus spike protein and the heparan sulfate proteoglycan (HSPG) (Clausen et al., 2020; Palacios‐Rápalo et al., 2021; Zhang et al., 2020). Another receptor, the toll‐like receptor 4 (TLR4), which interacts with the S1 subunit of spike protein, facilitates SARS‐CoV‐2 entry even in the absence of the ACE2 receptor (Aboudounya et al., 2021; Butnariu et al., 2021; Palacios‐Rápalo et al., 2021). To further elucidate the role of cholesterol in the life cycle of SARS‐CoV‐2, several studies have demonstrated that the manipulation of host membrane cholesterol alleviate virus entry into host cells. Indeed, it was demonstrated that depletion of membrane‐bound cholesterol from the lipid raft of ACE2‐rich cells impaired SARS‐CoV‐2 entry into host cells (Sanders et al., 2021).

Another component contributing to the clinical manifestations of COVID‐19 patients is calcium. A recent systematic review and meta‐analysis showed that coronary calcification is associated with increased mortality in patients with COVID‐19 (Cereda et al., 2022). This may be attributed to the greater activation, in these patients, of the renin‐angiotensin‐aldosterone system (RAAS), which increases the expression of ACE2 receptors on VSMC surface, thereby enhancing viral replication and vascular injury (Figure 1) (Beyerstedt et al., 2021; Cereda et al., 2022). This is in concordance with another study which showed that increased intracellular calcium load activates cathepsin L, which in turn aids in the cleavage of the spike S into S1 and S2 (Tang et al., 2020; Wei et al., 2022). S1 can bind to the ACE‐2 receptor, while S2 facilitates the endocytosis of the virus into the host cells (Pizzato et al., 2022; Wei et al., 2022). The binding of the S2 to the lipid raft microdomains of the target cell is enhanced by elevated Ca^2+^ levels (Sultan et al., 2022).

### 3.2 Role of calcium, cholesterol, and phosphate in VSMC phenotypic switch

**Figure 2:**
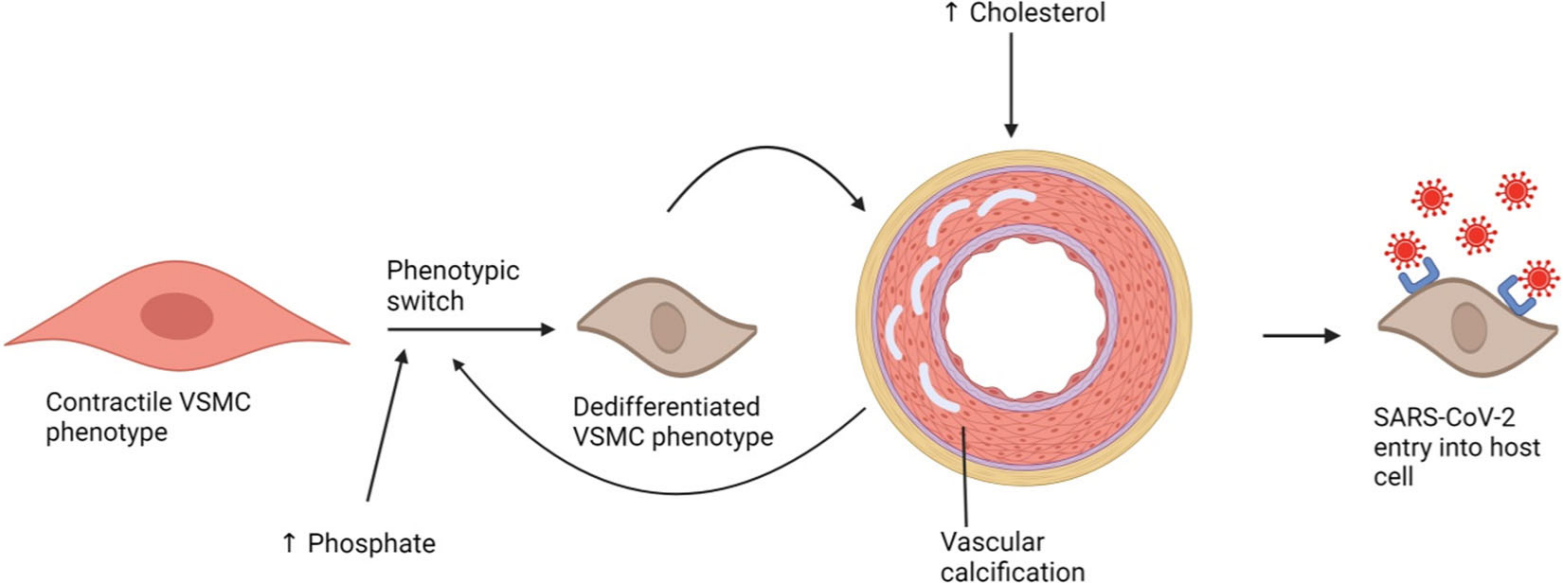
Summary of the interplay between vascular smooth muscle cell (VSMC) phenotypic switch, vascular calcification, and severe acute respiratory syndrome coronavirus 2 (SARS‐CoV‐2) entry into the cell. Contractile VSMCs can undergo a phenotypic switch under hyperphosphatemia, which results in dedifferentiated VSMCs. This can lead to vascular calcification, which can exert positive feedback on the phenotypic switch. Vascular calcification enhances the SARS‐CoV‐2 entry into the host cell, and can be stimulated by high cholesterol levels. Created with BioRender.com.

Increasing evidence suggests that one of the major precipitating factors of the COVID‐19 course is vascular calcification (Possari et al., 2021; Shabestari et al., 2022). Coronary artery calcification was associated with a worse prognosis in patients infected with SARS‐CoV‐2 (Dillinger et al., 2020; Luo et al., 2022; Shabestari et al., 2022). Moreover, many studies shed light on the higher mortality rate in COVID‐19 patients who had vascular calcification, in comparison to those without calcified vessels (Gupta et al., 2021; Meyer et al., 2023; Slipczuk et al., 2021). It is important to mention that vascular calcification mainly emerges from the osteo‐/chondrocyte phenotypic switch of VSMCs, which makes it highly essential to discuss the role of calcium, cholesterol, and phosphate on the VSMC phenotypic switch (Liu et al., 2022). Several studies show that the transient receptor potential vanilloid 4 (TRPV4) channel enables the entry of extracellular calcium (Figure 1) (Cao et al., 2018; Li, Gao, et al., 2021; Toft‐Bertelsen et al., 2019). This entry can result in neointimal hyperplasia and VSMC migration, while the L‐type voltage‐gated calcium channel has no effect on VSMC phenotype (Li, Gao, et al., 2021). Interestingly, medial calcification triggers changes in cell wall morphology and results in higher arterial stiffness (Jaminon et al., 2019), suggesting that this calcification is highly correlated with phenotypic switch of VSMCs where cells acquire some features of chondrocytes and osteoblasts (Jaminon et al., 2019) (Figure 2).

One of the factors affecting VSMC calcification, therefore promoting phenotypic switching, is cholesterol. A prospective study investigated this in 1980, with a follow‐up period of over 28 years (Armstrong et al., 2021). In each stage of development, mainly in adolescence, non‐HDL‐c was associated with coronary artery calcification (Armstrong et al., 2021). Moreover, low‐density lipoprotein particles (LDL‐P) were highly associated with the presence of coronary artery calcification, independently of other lipid particles (Prado et al., 2011; Zaid et al., 2016). In addition, the low‐density lipoprotein cholesterol (LDL‐C) was shown to be dependent on other LDL‐Ps and to be associated with coronary artery calcification, even if the association was weaker than that of LDL‐P (Figure 1) (Prado et al., 2011; Zaid et al., 2016).

Levels of phosphate are yet another factor that affects VSMC differentiation. Interestingly, increased inorganic phosphate (HiPO^4−^) levels rapidly activate the hypoxia‐inducible factor‐1 (HIF‐1), a transcription factor implicated in VSMC phenotype (Figure 1) (Mokas et al., 2016). HiPO^4‐^‐induced HIF‐1 activation drives expression of various genes like including vascular endothelial growth factor (VEGF), a critical angiogenic factor (Mokas et al., 2016; Zimna & Kurpisz, 2015). Interestingly, VEGF is expressed in osteoblast precursor cells that boost VSMC calcification and osteogenic trans‐differentiation (Figure 1) (Hu & Olsen, 2016; Mokas et al., 2016). Similarly, hyperphosphatemia in chronic kidney disease (CKD) increases the expression of β‐adrenergic receptors (Moser et al., 2021). Stimulation of these receptors activates the protein kinase A (PKA)/cyclic adenosine monophosphate (cAMP) response element‐binding protein (CREB) signaling pathway, which then enhances the osteogenic transdifferentiation of VSMCs (Figure 1) (Moser et al., 2021).

### 3.3 Effect of VSMC switch on calcification

Although the role of vascular calcification in promoting VSMC phenotypic switch is well established, some studies stated that these two processes may happen in a reversed order (Figure 2). For instance, VSMC phenotype switch was detected 4 weeks before arterial medial calcification (AMC) (Pai et al., 2011). Another study in ApoE^−/−^ mice reported that vascular remodeling results in the transdifferentiation of VSMCs, evident by increased migration and proliferation (Augstein et al., 2018). This switch was concomitant with increased expression of the SRY‐box transcription factor 9 (Sox9) (Augstein et al., 2018). Consequently, an increase in the number of apoptotic cells is noted, and ECM remodeling and increased calcium deposition ensue (Augstein et al., 2018; Faleeva et al., 2023) (Figure 1).

## 4 DEFENSE MECHANISMS OF THE PATHWAYS INVOLVING CALCIUM, PHOSPHATE, AND CHOLESTEROL

### 4.1 Inhibitors of SARS‐CoV‐2 entry into VSMCs

Inhibiting SARS‐CoV‐2 entry into host cells has been of much interest. A factor that plays an essential role in viral life cycle inhibition is lipid levels, especially at the stage of the virus fusion with the target cell membrane. While non‐HDL‐c is essential for VSMC calcification, allowing the SARS‐CoV‐2 to enter VSMCs more readily, HDL‐c has a markedly different effect. Indeed, as pertains to infection with this virus, multiple studies have shown that low levels of HDL‐c are associated with an increased risk of hospitalization, more severe disease, and even mortality (Lahoz et al., 2022; Zang et al., 2020). Among other studies, a cohort study involving half a million participants demonstrated the existence of this inversely proportional relation between HDL‐c level and the risk of SARS‐CoV2 infection (Lahoz et al., 2022; Zang et al., 2020).

**Figure 3:**
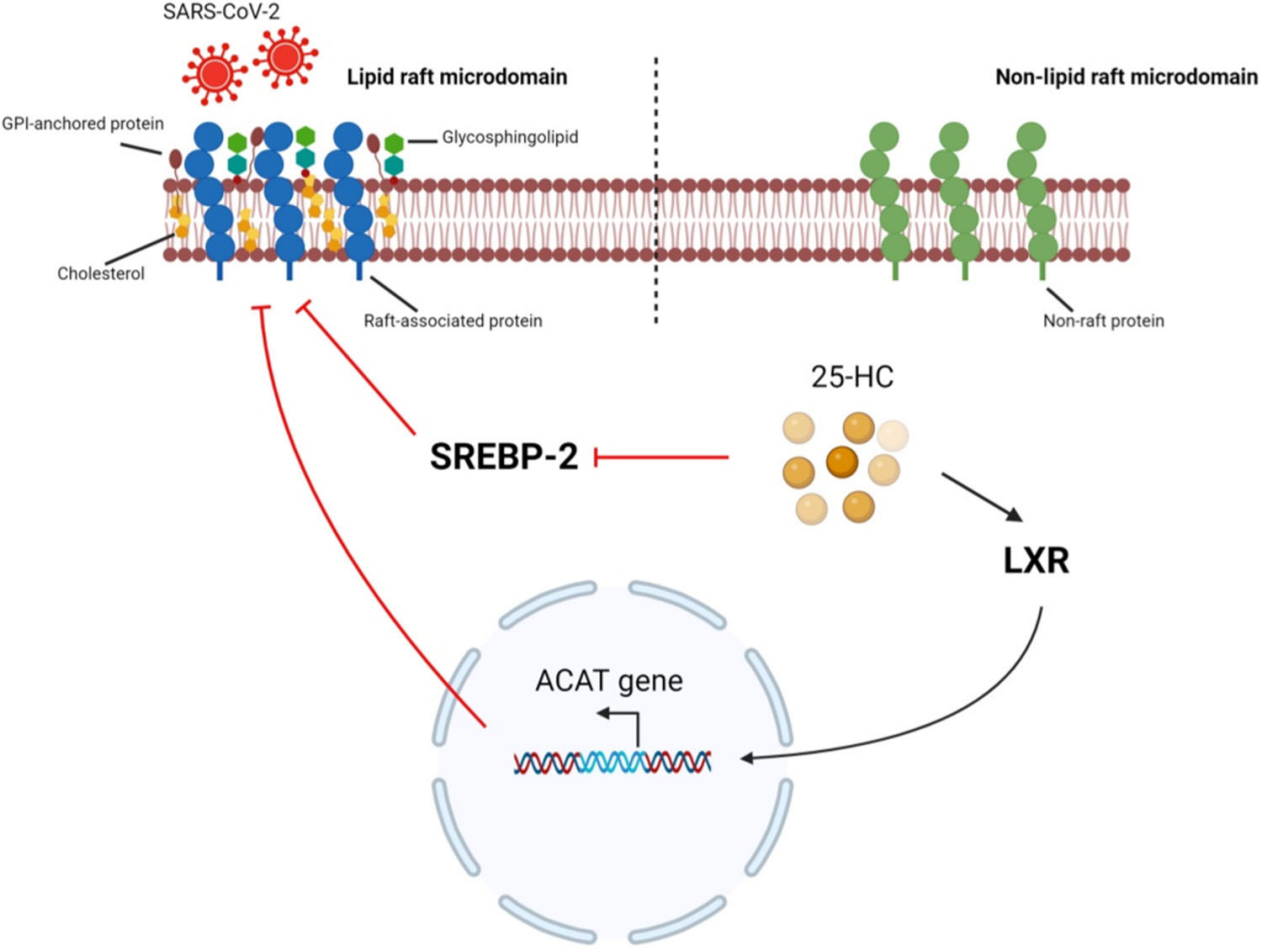
Mechanism by which 25HC blocks severe acute respiratory syndrome coronavirus 2 (SARS‐CoV‐2) fusion with the vascular smooth muscle cell (VSMC) lipid raft microdomain. 25‐hydroxycholesterol (25HC) inhibits sterol‐regulatory element binding proteins‐2 (SREBP‐2) and activates liver X receptor (LXR), which in turn stimulates acyl‐CoA cholesterol acyltransferase. This results in the depletion of cholesterol, glycosphingolipids, and the glycosylphosphatidylinositol (GPI)‐anchored proteins from the lipid raft microdomain, which then inhibits the entry of the SARS‐CoV‐2 into the host cell. Created with BioRender.com. ACAT, acyl‐CoA cholesterol acyltransferase.

The role of cholesterol role in inhibiting coronavirus entry is also related to another pathway involving 25‐hydroxycholesterol (25HC) (Kočar et al., 2021). This 25HC, the product of cholesterol‐25‐hydroxylase (CH25H), can block the sterol‐regulatory element binding protein‐2 (SREBP2), and can stimulate the liver X receptor (LXR), hence activating the Acyl‐coenzyme A: cholesterol acyl‐transferase (Figure 3) (Kočar et al., 2021; Mao et al., 2022). These actions repress the cholesterol and the lipid raft microdomains present in the cell membrane, thus blocking membrane fusion and disrupting viral protein maturation (Figure 3) (Kočar et al., 2021; Mao et al., 2022; Zang et al., 2020). Supportably, 25HC was found to suppress SARS‐CoV‐2's infection in lung epithelial cells (Wang et al., 2020). Since 25HC is an endogenous particle with unrecognized toxicity at adequate concentrations, it is considered an effective therapeutic substance for COVID‐19 (Wang et al., 2020). Multiple studies in recent years have demonstrated the broad‐spectrum antiviral activity of 25HC by virtue of its ability to suppress SARS‐CoV‐2 spike protein‐catalyzed membrane fusion, thus viral entry into VSMCs.

Entry of SARS‐CoV‐2 may also be inhibited by 27‐hydroxycholesterol (27OHC). 27OHC exerts an antiviral effect against many viruses, including two human CoVs belonging to the β‐coronavirus genus: SARS‐CoV‐2 and *Human Coronavirus Organ Culture 43* (HcoV‐OC43) (Mao et al., 2022; Marcello et al., 2020). It has been suggested that 27OHC acts by modifying the cell structure rather than by targeting viral components (Marcello et al., 2020). This mode of action makes the 27OHC able to impede SARS‐CoV‐2 entry into cells including VSMCs, and hence rendering it as a potentially potent inhibitor.

Another process involved in inhibiting coronavirus entry is ACE2 shedding. As mentioned earlier, the ACE2 catalytic ectodomain is a required entry receptor for SARS‐CoV‐2 infection (García‐Escobar et al., 2022; Glende et al., 2008). The liberation process of the ACE2 catalytic domain is facilitated by disintegrin and metalloproteinase domain‐containing protein 17 (ADAM17) through the calcium signaling pathway field (García‐Escobar et al., 2022; Zipeto et al., 2020). This mechanism “releases” a soluble catalytic ectodomain of ACE2 (García‐Escobar et al., 2022). Such soluble forms of ACE2 block SARS‐CoV‐2 infection, thus offering a prognosis improvement in COVID‐19 patients (García‐Escobar et al., 2022; Glende et al., 2008; Haga et al., 2010; Zipeto et al., 2020). The shedding of the ACE2 catalytic ectodomain is a predictor of all‐cause death, including cardiovascular mortality, cardiac remodeling, and endothelial dysfunction (García‐Escobar et al., 2022; Haga et al., 2010).

### 4.2 Inhibitors of calcification or VSMC switch

As stated above, vascular calcification and VSMC phenotypic switch affect SARS‐CoV‐2 entry (Figure 2). Therefore, it is not surprising that efforts are being expended to determine whether inhibitors of calcification could be efficacious against COVID‐19. Contextually, patients with low calcium scores are more resilient to the SARS‐CoV‐2 infection (Cereda et al., 2022). Similarly, other studies confirm the antiviral effect of calcium channel blockers especially against coronaviruses, and further suggest their potential in suppressing viral entry as well as treating SARS‐CoV‐2‐infected patients (Crespi & Alcock, 2021; Fani et al., 2023; Straus et al., 2021).

Calcification and phenotypic switch of VSMCs can be modulated by various enzymes. One such enzyme is superoxide dismutase 2 (SOD2), which downregulates mitochondrial reactive oxygen species, resulting in the downregulation of Ca^2+^‐sensitive intracellular cysteine protease calpain‐1 (Roman‐Garcia et al., 2011; Tsai et al., 2021). This leads to lower expressions of alkaline phosphatase and increases the expression of adenosine 5′‐triphosphate (ATP) synthases, as well as calcification inhibitors (Roman‐Garcia et al., 2011; Tsai et al., 2021). This, in turn, leads to diminished VSMC apoptosis and a low phosphate‐induced VSMC calcification, thereby inhibiting VSMC phenotype switch (Roman‐Garcia et al., 2011; Tsai et al., 2021).

Some endogenous molecules may also be involved in this phenotypic switch. For instance, ATP, uridine‐5′‐triphosphate (UTP), and the ubiquitous mineralization inhibitor pyrophosphate (Ppi) can protect VSMCs from apoptosis (Opdebeeck et al., 2020; Patel et al., 2018). As such, those cells will no longer create a nucleation site for the hydroxyapatite crystal formation (Patel et al., 2018), hence preventing VSMC calcification from taking place (Patel et al., 2018). In addition, calcium and phosphate play an essential role in the VSMC phenotypic switch. These two minerals induce vascular calcification (Freise et al., 2015), which triggers VSMC dedifferentiation into a synthetic osteoblast‐like phenotype (Houben et al., 2016; Opdebeeck et al., 2020). However, the coexistence of calcium and phosphate in large quantities yields a feedback loop; they upregulate Matrix Gla protein (MGP) levels, and in turn block calcification (Houben et al., 2016).

## 5 COMORBIDITIES, VSMC, AND COVID‐19

### 5.1 CKD and COVID‐19

Several studies highlight the notion that CKD may increase the risk of COVID‐19 (Schiffl & Lang, 2023). In patients with renal failure, the immune system is suboptimal, and a decrease in important immunologic mediators such as antibodies and complement is noted (Schiffl & Lang, 2023). This implies a consequent decline in the innate and adaptive immune system efficiency, resulting in more susceptibility to infections.

**Figure 4:**
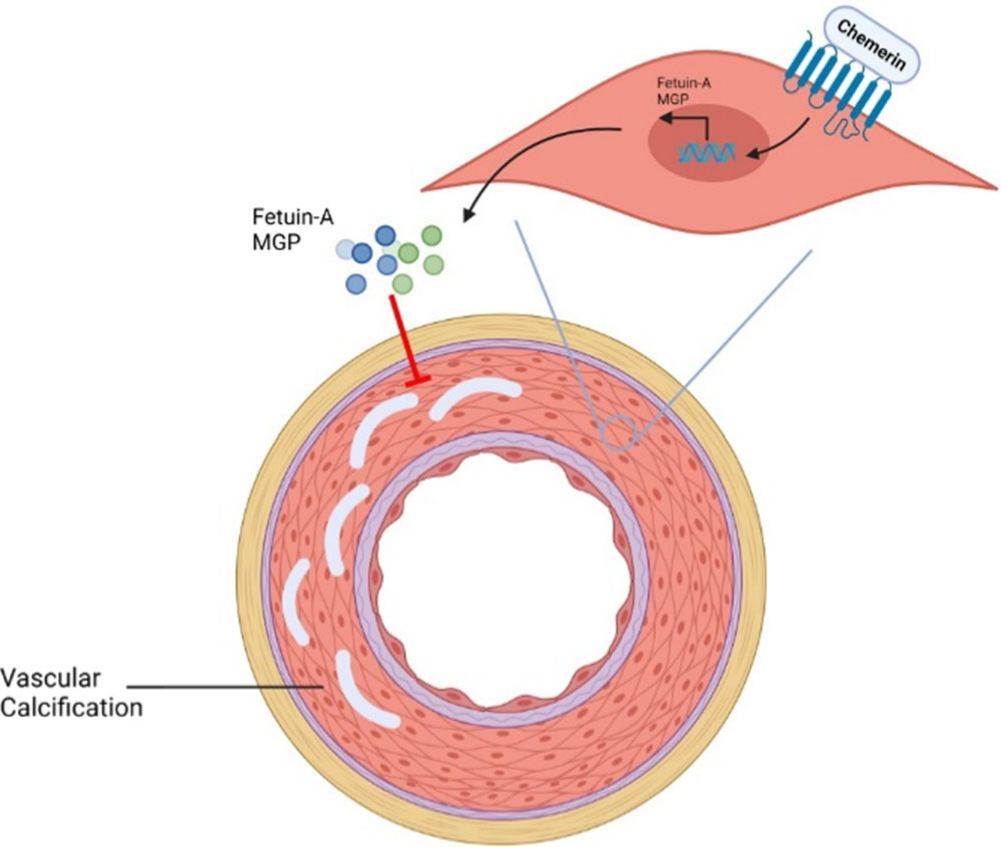
The mechanism of inhibiting calcification through chemerin's effect on vascular smooth muscle cells (VSMC). Chemerin, an adipokine found in patients with impaired renal function binds to its receptor on VSMC surface, promoting in the expression of two calcification inhibitors, the fetuin A and matrix Gla protein (MGP). Created with BioRender.com.

Because lower calcium levels may put a brake on SARS‐CoV‐2 entry, abating vascular calcification in CKD patient can potentially reduce their risk of being infected with SARS‐CoV‐2 (Jdiaa et al., 2022). In this context, interactions between VSMCs and chemerin, a biomarker of declined renal function, have been noted to play a role in calcification (Figure 4) (Carracedo et al., 2019; Su et al., 2021). Indeed, chemerin binds to its G‐protein coupled receptor on VSMCs. This triggers a signaling pathway that culminates in increased expression of two calcification inhibitors, namely fetuin‐A and MGP (Carracedo et al., 2019; Sun et al., 2021). Both these proteins suppress osteogenic differentiation of VSMCs, and hence reduce vascular calcification (Figure 4) (Carracedo et al., 2019).

### 5.2 Atherosclerosis and COVID‐19

**Figure 5:**
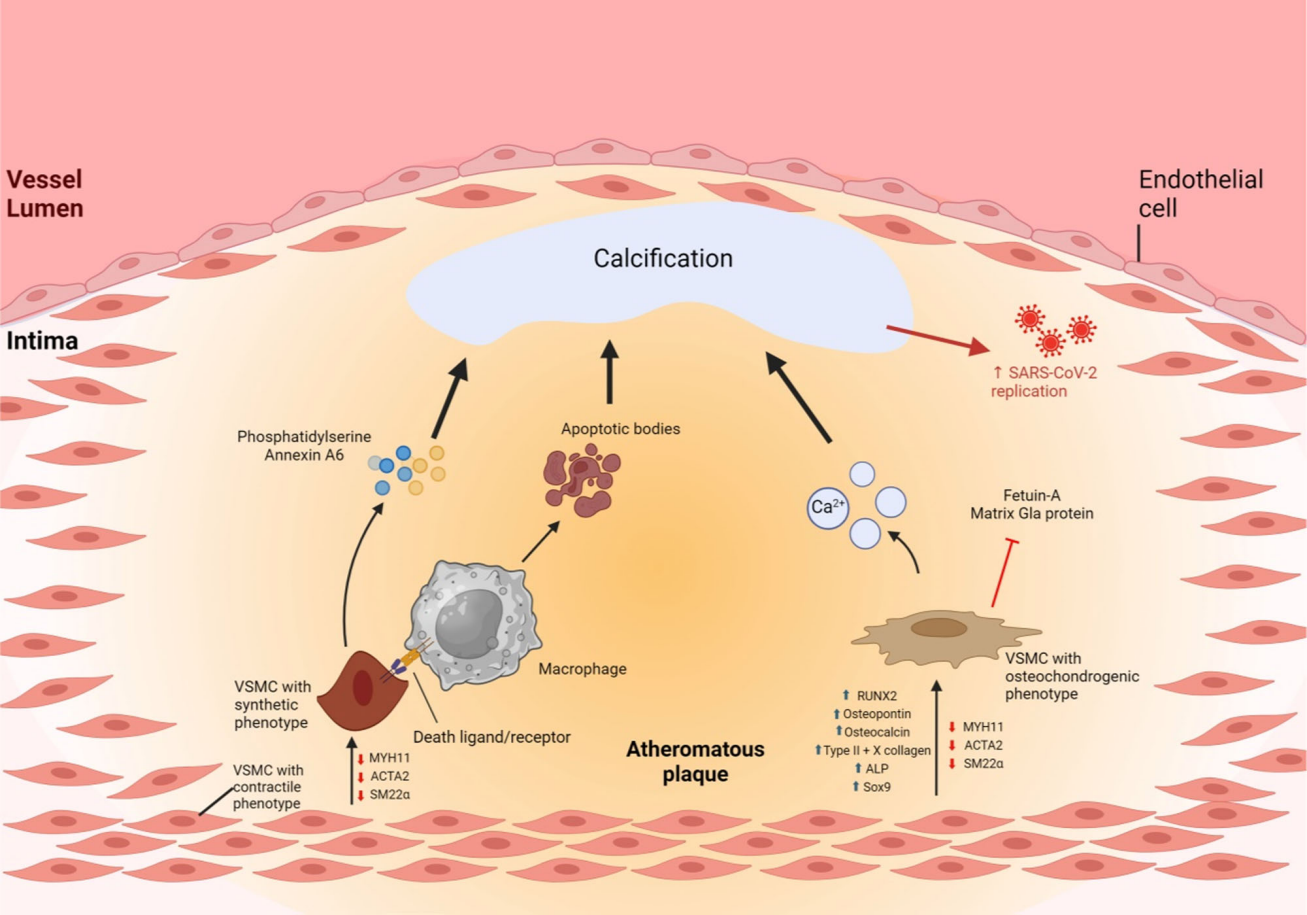
The effect of various vascular smooth muscle cell (VSMC) phenotypes on the calcification state of the atherosclerotic plaque. Some contractile VSMCs' markers, such as myosin heavy chain (MYH11), smooth muscle aortic alpha‐actin (ACTA2), and smooth muscle 22 alpha (SM22α) are downregulated in atherosclerosis; however, others like runt‐related transcription factor 2 (RUNX2), osteopontin, osteocalcin, type II and X collagen, alkaline phosphatase (ALP), and SRY‐box transcription factor 9 (Sox9) are upregulated. This prompts VSMCs to assume another phenotype. Synthetic VSMCs secrete PS and annexin A6, recruit macrophages to the plaque, and decrease calcification inhibitors like matrix Gla protein (MGP) and fetuin‐A, therefore inducing in calcium deposits. Created with BioRender.com.

Atherosclerosis, a major cardiovascular disease, is the most common cause of worldwide mortalities. It is characterized by the formation of vascular plaques made up of fats, cholesterol, fibrin, among others, and it also involves VSMC phenotypic switching (Bennett et al., 2016; Ibrahim et al., 2023). Indeed, contractile phenotype markers like smooth muscle cell myosin heavy chain (MYH11), and smooth muscle aortic alpha‐actin (ACTA2), SMC lineage‐restricted protein are reduced in atherosclerosis (Bennett et al., 2016). This downregulation is concomitant with increased VSMC‐derived secretion of exosomes carrying molecules, like phosphatidylserine or annexin A6, that induce calcification in the atherosclerotic plaque (Bennett et al., 2016; Grootaert & Bennett, 2021). Furthermore, production of membrane‐bound apoptotic bodies in the plaque ensue (Bennett et al., 2016; Grootaert & Bennett, 2021). These bodies induce the recruitment of macrophages to this plaque, making it a nucleation site for the calcification (Figure 5) (Bennett et al., 2016; Grootaert & Bennett, 2021).

In the context of atherosclerosis, VSMCs may follow another dedifferentiation path. For instance, they may switch to an osteochondrogenic phenotype (Sanyour et al., 2020) by virtue of a notable suppression of smooth muscle 22 alpha (SM22α) along with the upregulation of osteochondrogenic markers such as runt‐related transcription factor 2 (RUNX2), osteopontin, osteocalcin, Type II and X collagen, alkaline phosphatase, and Sox9, hence precipitating calcification (Grootaert & Bennett, 2021). Another cause for this calcification in atherosclerotic patients is the deposition of calcifying vesicles, and the downregulation of mineralization inhibitory molecules such as vit K‐dependent MGP and fetuin‐A (Figure 5) (Durham et al., 2018; Grootaert & Bennett, 2021). Consequently, microcalcification in the fibrous cap and macrocalcification in the necrotic core of the plaque are induced (Grootaert & Bennett, 2021). Given that vascular calcification is a player in the pathogenesis of COVID‐19, patients suffering from atherosclerosis, especially ones with calcified plaques, are at higher risk of suffering from COVID‐19 or acquiring its severe symptoms (Poznyak et al., 2021).

## 6 CONCLUSION

The interplay between VSMCs phenotypic switch and cholesterol, calcium, or phosphate is crucial for the pathogenesis of SARS‐CoV2 or its remission, especially in patients with CKD and atherosclerosis. That would be especially important, as it represents a potential for targeting viral entry inhibition, thus antiviral therapy, since currently, many studies aim at targeting pathways that mediate viral entry. Furthermore, despite the currently available therapeutic options, more studies are needed to identify molecules other than cholesterol, calcium, and phosphate that could prove to be tractable targets in the fight against this virus (Bhimraj et al., 2024; Giordo et al., 2021; Issa et al., 2021; Kaddoura et al., 2020; Wehbe, Wehbe, et al., 2021; Younis et al., 2020, 2021; Zareef et al., 2020). This will aid in developing new drugs that can treat patients who are resistant to the current standard of care.

## AUTHOR CONTRIBUTIONS

**Laura Ghanem**: Writing—original draft. **Dina Essayli**: Writing—original draft. **Jana Kotaich**: Writing—original draft. **Mohammad Al Zein**: Writing—original draft. **Amirhossein Sahebkar**: Writing—original draft; writing—review and editing. **Ali H. Eid**: Writing—review and editing; supervision; resources; conceptualization.

## CONFLICT OF INTEREST STATEMENT

The authors declare no conflict of interest.

## References

[jcp31424-bib-0001] Aboudounya, M. M. , Holt, M. R. , & Heads, R. J. (2021). SARS‐CoV‐2 spike S1 glycoprotein is a TLR4 agonist, upregulates ACE2 expression and induces pro‐inflammatory M1 macrophage polarisation. BioRxiv, 2011, 455921.

[jcp31424-bib-0002] Armstrong, M. K. , Fraser, B. J. , Hartiala, O. , Buscot, M.‐J. , Juonala, M. , Wu, F. , Koskinen, J. , Hutri‐Kähönen, N. , Kähönen, M. , Laitinen, T. P. , Lehtimäki, T. , Viikari, J. S. A. , Raitakari, O. T. , & Magnussen, C. G. (2021). Association of non–high‐density lipoprotein cholesterol measured in adolescence, young adulthood, and mid‐adulthood with coronary artery calcification measured in mid‐adulthood. JAMA Cardiology, 6(6), 661–668. 33502454 10.1001/jamacardio.2020.7238 PMC7841578

[jcp31424-bib-0003] Augstein, A. , Mierke, J. , Poitz, D. M. , & Strasser, R. H. (2018). Sox9 is increased in arterial plaque and stenosis, associated with synthetic phenotype of vascular smooth muscle cells and causes alterations in extracellular matrix and calcification. Biochimica et Biophysica Acta (BBA)‐Molecular Basis of Disease, 1864(8), 2526–2537. 29777903 10.1016/j.bbadis.2018.05.009

[jcp31424-bib-0004] Bennett, M. R. , Sinha, S. , & Owens, G. K. (2016). Vascular smooth muscle cells in atherosclerosis. Circulation Research, 118(4), 692–702. 26892967 10.1161/CIRCRESAHA.115.306361 PMC4762053

[jcp31424-bib-0005] Beyerstedt, S. , Casaro, E. B. , & Rangel, É. B. (2021). COVID‐19: Angiotensin‐converting enzyme 2 (ACE2) expression and tissue susceptibility to SARS‐CoV‐2 infection. European Journal of Clinical Microbiology & Infectious Diseases, 40, 905–919. 33389262 10.1007/s10096-020-04138-6 PMC7778857

[jcp31424-bib-0006] Bhimraj, A. , Morgan, R. L. , Shumaker, A. H. , Baden, L. R. , Cheng, V. C. C. , Edwards, K. M. , Gallagher, J. C. , Gandhi, R. T. , Muller, W. J. , Nakamura, M. M. , O'Horo, J. C. , Shafer, R. W. , Shoham, S. , Murad, M. H. , Mustafa, R. A. , Sultan, S. , & Falck‐Ytter, Y. (2024). Infectious Diseases Society of America Guidelines on the Treatment and Management of Patients with COVID‐19 (September 2022). Clinical Infectious Diseases, 78(7), e250–e349. 10.1093/cid/ciac724 36063397 PMC9494372

[jcp31424-bib-0007] Bikdeli, B. , Madhavan, M. V. , Jimenez, D. , Chuich, T. , Dreyfus, I. , Driggin, E. , Nigoghossian, C. D. , Ageno, W. , Madjid, M. , Guo, Y. , Tang, L. V. , Hu, Y. , Giri, J. , Cushman, M. , Quéré, I. , Dimakakos, E. P. , Gibson, C. M. , Lippi, G. , Favaloro, E. J. , … Lip, G. Y. H. (2020). COVID‐19 and thrombotic or thromboembolic disease: Implications for prevention, antithrombotic therapy, and follow‐up. Journal of the American College of Cardiology, 75(23), 2950–2973. 10.1016/j.jacc.2020.04.031 32311448 PMC7164881

[jcp31424-bib-0008] Bundy, K. , Boone, J. , & Simpson, C. L. (2021). Wnt signaling in vascular calcification. Frontiers in Cardiovascular Medicine, 8, 708470. 34595218 10.3389/fcvm.2021.708470 PMC8476789

[jcp31424-bib-0009] Butnariu, A.‐B. , Look, A. , Grillo, M. , Tabish, T. A. , McGarvey, M. J. , & Pranjol, M. Z. I. (2021). SARS‐CoV‐2–host cell surface interactions and potential antiviral therapies. Interface Focus, 12(1), 20200081. 34956606 10.1098/rsfs.2020.0081 PMC8662392

[jcp31424-bib-0010] Cao, S. , Anishkin, A. , Zinkevich, N. S. , Nishijima, Y. , Korishettar, A. , Wang, Z. , Fang, J. , Wilcox, D. A. , & Zhang, D. X. (2018). Transient receptor potential vanilloid 4 (TRPV4) activation by arachidonic acid requires protein kinase A–mediated phosphorylation. Journal of Biological Chemistry, 293(14), 5307–5322. 29462784 10.1074/jbc.M117.811075 PMC5892583

[jcp31424-bib-0011] Carracedo, M. , Witasp, A. , Qureshi, A. R. , Laguna‐Fernandez, A. , Brismar, T. , Stenvinkel, P. , & Bäck, M. (2019). Chemerin inhibits vascular calcification through ChemR23 and is associated with lower coronary calcium in chronic kidney disease. Journal of Internal Medicine, 286(4), 449–457. 31197872 10.1111/joim.12940 PMC6852438

[jcp31424-bib-0012] Casari, I. , Manfredi, M. , Metharom, P. , & Falasca, M. (2021). Dissecting lipid metabolism alterations in SARS‐CoV‐2. Progress in Lipid Research, 82, 101092. 33571544 10.1016/j.plipres.2021.101092 PMC7869689

[jcp31424-bib-0013] Cereda, A. , Allievi, L. , Palmisano, A. , Tumminello, G. , Barbieri, L. , Mangieri, A. , Laricchia, A. , Khokhar, A. , Giannini, F. , Toselli, M. , Sangiorgi, G. M. , Esposito, A. , Aseni, P. , Lucreziotti, S. , Mafrici, A. , & Carugo, S. (2022). Systematic review and meta‐analysis on coronary calcifications in COVID‐19. Emergency Radiology, 29(4), 631–643. 10.1007/s10140-022-02048-y 35501615 PMC9059910

[jcp31424-bib-0014] Chatzis, D. G. , Magounaki, K. , Pantazopoulos, I. , & Bhaskar, S. M. M. (2022). COVID‐19 and the cardiovascular system‐current knowledge and future perspectives. World Journal of Clinical Cases, 10(27), 9602–9610. 10.12998/wjcc.v10.i27.9602 36186205 PMC9516937

[jcp31424-bib-0015] Chilazi, M. , Duffy, E. Y. , Thakkar, A. , & Michos, E. D. (2021). COVID and cardiovascular disease: What we know in 2021. Current Atherosclerosis Reports, 23(7), 37. 10.1007/s11883-021-00935-2 33983522 PMC8117457

[jcp31424-bib-0016] Clausen, T. M. , Sandoval, D. R. , Spliid, C. B. , Pihl, J. , Perrett, H. R. , Painter, C. D. , Narayanan, A. , Majowicz, S. A. , Kwong, E. M. , McVicar, R. N. , Thacker, B. E. , Glass, C. A. , Yang, Z. , Torres, J. L. , Golden, G. J. , Bartels, P. L. , Porell, R. N. , Garretson, A. F. , Laubach, L. , … Esko, J. D. (2020). SARS‐CoV‐2 infection depends on cellular heparan sulfate and ACE2. Cell, 183(4), 1043–1057.e15. 32970989 10.1016/j.cell.2020.09.033 PMC7489987

[jcp31424-bib-0017] Clerkin, K. J. , Fried, J. A. , Raikhelkar, J. , Sayer, G. , Griffin, J. M. , Masoumi, A. , Jain, S. S. , Burkhoff, D. , Kumaraiah, D. , Rabbani, L. , Schwartz, A. , & Uriel, N. (2020). COVID‐19 and cardiovascular disease. Circulation, 141(20), 1648–1655. 10.1161/circulationaha.120.046941 32200663

[jcp31424-bib-0018] Crespi, B. , & Alcock, J. (2021). Conflicts over calcium and the treatment of COVID‐19. Evolution, Medicine, and Public Health, 9(1), 149–156. 10.1093/emph/eoaa046 33732462 PMC7717197

[jcp31424-bib-0019] Deng, Y. , Li, S. , Chen, Z. , Wang, W. , Geng, B. , & Cai, J. (2021). Mdivi‐1, a mitochondrial fission inhibitor, reduces angiotensin‐II‐induced hypertension by mediating VSMC phenotypic switch. Biomedicine & Pharmacotherapy = Biomedecine & Pharmacotherapie, 140, 111689. 34004510 10.1016/j.biopha.2021.111689

[jcp31424-bib-0020] Dillinger, J. G. , Benmessaoud, F. A. , Pezel, T. , Voicu, S. , Sideris, G. , Chergui, N. , Hamzi, L. , Chauvin, A. , Leroy, P. , Gautier, J. F. , Sène, D. , & Henry, P. (2020). Coronary artery calcification and complications in patients with COVID‐19. JACC Cardiovascular Imaging, 13(11), 2468–2470. 10.1016/j.jcmg.2020.07.004 33153535 PMC7605736

[jcp31424-bib-0021] Durham, A. L. , Speer, M. Y. , Scatena, M. , Giachelli, C. M. , & Shanahan, C. M. (2018). Role of smooth muscle cells in vascular calcification: Implications in atherosclerosis and arterial stiffness. Cardiovascular Research, 114(4), 590–600. 29514202 10.1093/cvr/cvy010 PMC5852633

[jcp31424-bib-0022] Faleeva, M. , Ahmad, S. , Lynham, S. , Watson, G. , Whitehead, M. , Cox, S. , & Shanahan, C. M. (2023). Sox9 accelerates vascular ageing by regulating extracellular matrix composition and stiffness. bioRxiv, 2023, 539285. 10.1161/CIRCRESAHA.123.323365 PMC10826924 38179698

[jcp31424-bib-0023] Fani, M. , Moossavi, M. , Bakhshi, H. , Jahrodi, A. N. , Khazdair, M. R. , Zardast, A. H. , & Ghafari, S. (2023). Targeting host calcium channels and viroporins: A promising strategy for SARS‐CoV‐2 therapy. Future Virology, 18, 797–807. 10.2217/fvl-2022-0203 PMC10494978 37700758

[jcp31424-bib-0024] Freise, C. , Kim, K. Y. , & Querfeld, U. (2015). A Lindera obtusiloba extract blocks calcium‐/phosphate‐induced transdifferentiation and calcification of vascular smooth muscle cells and interferes with matrix metalloproteinase‐2 and metalloproteinase‐9 and NF‐B. Evidence‐Based Complementary and Alternative Medicine, 2015, 1–8. 10.1155/2015/679238 PMC4534752 26294927

[jcp31424-bib-0025] Frismantiene, A. , Philippova, M. , Erne, P. , & Resink, T. J. (2018). Smooth muscle cell‐driven vascular diseases and molecular mechanisms of VSMC plasticity. Cellular Signalling, 52, 48–64. 30172025 10.1016/j.cellsig.2018.08.019

[jcp31424-bib-0026] Fu, L. , Liu, X. , Su, Y. , Ma, J. , & Hong, K. (2021). Prevalence and impact of cardiac injury on COVID‐19: A systematic review and meta‐analysis. Clinical Cardiology, 44(2), 276–283. 10.1002/clc.23540 33382482 PMC7852167

[jcp31424-bib-0027] Furmanik, M. , Chatrou, M. , van Gorp, R. , Akbulut, A. , Willems, B. , Schmidt, H. , van Eys, G. , Bochaton‐Piallat, M. L. , Proudfoot, D. , Biessen, E. , Hedin, U. , Perisic, L. , Mees, B. , Shanahan, C. , Reutelingsperger, C. , & Schurgers, L. (2020). Reactive oxygen‐forming Nox5 links vascular smooth muscle cell phenotypic switching and extracellular vesicle‐mediated vascular calcification. Circulation Research, 127(7), 911–927. 32564697 10.1161/CIRCRESAHA.119.316159

[jcp31424-bib-0028] Gao, L. , Chen, Q. , Zhou, X. , & Fan, L. (2012). The role of hypoxia‐inducible factor 1 in atherosclerosis. Journal of Clinical Pathology, 65(10), 872–876. 10.1136/jclinpath-2012-200828 22569539

[jcp31424-bib-0029] García‐Escobar, A. , Vera‐Vera, S. , Jurado‐Román, A. , Jiménez‐Valero, S. , Galeote, G. , & Moreno, R. (2022). Calcium signaling pathway is involved in the shedding of ACE2 catalytic ectodomain: New insights for clinical and therapeutic applications of ACE2 for COVID‐19. Biomolecules, 12(1), 76. 10.3390/biom12010076 35053224 PMC8774087

[jcp31424-bib-0030] Giordo, R. , Zinellu, A. , Eid, A. H. , & Pintus, G. (2021). Therapeutic potential of resveratrol in COVID‐19‐associated hemostatic disorders. Molecules, 26(4), 856. 10.3390/molecules26040856 33562030 PMC7915700

[jcp31424-bib-0031] Glende, J. , Schwegmann‐Wessels, C. , Al‐Falah, M. , Pfefferle, S. , Qu, X. , Deng, H. , Drosten, C. , Naim, H. Y. , & Herrler, G. (2008). Importance of cholesterol‐rich membrane microdomains in the interaction of the S protein of SARS‐coronavirus with the cellular receptor angiotensin‐converting enzyme 2. Virology, 381(2), 215–221. 18814896 10.1016/j.virol.2008.08.026 PMC7103374

[jcp31424-bib-0032] Grootaert, M. O. J. , & Bennett, M. R. (2021). Vascular smooth muscle cells in atherosclerosis: Time for a re‐assessment. Cardiovascular Research, 117(11), 2326–2339. 33576407 10.1093/cvr/cvab046 PMC8479803

[jcp31424-bib-0033] Guan, W. , Ni, Z. , Hu, Y. , Liang, W. , Ou, C. , He, J. , Liu, L. , Shan, H. , Lei, C. , Hui, D. S. C. , Du, B. , Li, L. , Zeng, G. , Yuen, K. Y. , Chen, R. , Tang, C. , Wang, T. , Chen, P. , Xiang, J. , … Zhong, N. (2020). Clinical characteristics of coronavirus disease 2019 in China. New England Journal of Medicine, 382(18), 1708–1720. 10.1056/NEJMoa2002032 32109013 PMC7092819

[jcp31424-bib-0034] Gupta, Y. S. , Finkelstein, M. , Manna, S. , Toussie, D. , Bernheim, A. , Little, B. P. , Concepcion, J. , Maron, S. Z. , Jacobi, A. , Chung, M. , Kukar, N. , Voutsinas, N. , Cedillo, M. A. , Fernandes, A. , Eber, C. , Fayad, Z. A. , & Hota, P. (2021). Coronary artery calcification in COVID‐19 patients: An imaging biomarker for adverse clinical outcomes. Clinical Imaging, 77, 1–8. 10.1016/j.clinimag.2021.02.016 33601125 PMC7875715

[jcp31424-bib-0035] Haga, S. , Nagata, N. , Okamura, T. , Yamamoto, N. , Sata, T. , Yamamoto, N. , Sasazuki, T. , & Ishizaka, Y. (2010). TACE antagonists blocking ACE2 shedding caused by the spike protein of SARS‐CoV are candidate antiviral compounds. Antiviral Research, 85(3), 551–555. 19995578 10.1016/j.antiviral.2009.12.001 PMC7114272

[jcp31424-bib-0036] Harrison, A. G. , Lin, T. , & Wang, P. (2020). Mechanisms of SARS‐CoV‐2 transmission and pathogenesis. Trends in Immunology, 41(12), 1100–1115. 33132005 10.1016/j.it.2020.10.004 PMC7556779

[jcp31424-bib-0037] Harrison, S. L. , Buckley, B. , Rivera‐Caravaca, J. M. , Zhang, J. , & Lip, G. (2021). Cardiovascular risk factors, cardiovascular disease, and COVID‐19: An umbrella review of systematic reviews. European Heart Journal. Quality of Care & Clinical Outcomes, 7(4), 330–339. 10.1093/ehjqcco/qcab029 34107535 PMC8294691

[jcp31424-bib-0038] Helms, J. , Combes, A. , & Aissaoui, N. (2022). Cardiac injury in COVID‐19. Intensive Care Medicine, 48(1), 111–113. 10.1007/s00134-021-06555-3 34727214 PMC8562019

[jcp31424-bib-0039] Hessami, A. , Shamshirian, A. , Heydari, K. , Pourali, F. , Alizadeh‐Navaei, R. , Moosazadeh, M. , Abrotan, S. , Shojaie, L. , Sedighi, S. , Shamshirian, D. , & Rezaei, N. (2021). Cardiovascular diseases burden in COVID‐19: Systematic review and meta‐analysis. The American Journal of Emergency Medicine, 46, 382–391. 10.1016/j.ajem.2020.10.022 33268238 PMC7561581

[jcp31424-bib-0040] Houben, E. , Neradova, A. , Schurgers, L. , & Vervloet, M. V. M. (2016). L'influenza del fosforo, del calcio e del magnesio sulla proteina Gla di matrice e sul processo di calcificazione vascolare: Una review sistematica. Giornale Italiano di Nefrologia: Organo Ufficiale Della Società Italiana di Nefrologia, 33(6), 1724–5590. 28134400

[jcp31424-bib-0041] Hu, K. , & Olsen, B. R. (2016). Osteoblast‐derived VEGF regulates osteoblast differentiation and bone formation during bone repair. Journal of Clinical Investigation, 126(2), 509–526. 26731472 10.1172/JCI82585 PMC4731163

[jcp31424-bib-0042] Ibrahim, M. , Suleiman, M. E. , Gandomkar, Z. , Tavakoli Taba, A. , Arnott, C. , Jorm, L. , Barraclough, J. Y. , Barbieri, S. , & Brennan, P. C. (2023). Associations of breast arterial calcifications with cardiovascular disease. Journal of Women's Health, 32(5), 529–545. 10.1089/jwh.2022.0394 36930147

[jcp31424-bib-0043] Issa, H. , Eid, A. H. , Berry, B. , Takhviji, V. , Khosravi, A. , Mantash, S. , Nehme, R. , Hallal, R. , Karaki, H. , Dhayni, K. , Faour, W. H. , Kobeissy, F. , Nehme, A. , & Zibara, K. (2021). Combination of angiotensin (1‐7) agonists and convalescent plasma as a new strategy to overcome angiotensin converting enzyme 2 (ACE2) inhibition for the treatment of COVID‐19. Frontiers in Medicine, 8:620990. 10.3389/fmed.2021.620990 33816521 PMC8012486

[jcp31424-bib-0044] Jaminon, A. , Reesink, K. , Kroon, A. , & Schurgers, L. (2019). The role of vascular smooth muscle cells in arterial remodeling: Focus on calcification‐related processes. International Journal of Molecular Sciences, 20(22), 5694. 10.3390/ijms20225694 31739395 PMC6888164

[jcp31424-bib-0045] Jdiaa, S. S. , Mansour, R. , El Alayli, A. , Gautam, A. , Thomas, P. , & Mustafa, R. A. (2022). COVID‐19 and chronic kidney disease: An updated overview of reviews. Journal of Nephrology, 35(1), 69–85. 10.1007/s40620-021-01206-8 35013985 PMC8747880

[jcp31424-bib-0046] Kaddoura, M. , AlIbrahim, M. , Hijazi, G. , Soudani, N. , Audi, A. , Alkalamouni, H. , Haddad, S. , Eid, A. , & Zaraket, H. (2020). COVID‐19 therapeutic options under investigation. Frontiers in Pharmacology, 11, 1196. 10.3389/fphar.2020.01196 32848795 PMC7424051

[jcp31424-bib-0047] Kar, M. (2022). Vascular dysfunction and its cardiovascular consequences during and after COVID‐19 infection: A narrative review. Vascular Health and Risk Management, 18, 105–112. 10.2147/vhrm.s355410 35283631 PMC8906855

[jcp31424-bib-0048] Kočar, E. , Režen, T. , & Rozman, D. (2021). Cholesterol, lipoproteins, and COVID‐19: Basic concepts and clinical applications. Biochimica et Biophysica Acta (BBA)‐Molecular and Cell Biology of Lipids, 1866(2), 158849. 33157278 10.1016/j.bbalip.2020.158849 PMC7610134

[jcp31424-bib-0049] Lahoz, C. , Salinero‐Fort, M. A. , Cárdenas, J. , Rodríguez‐Artalejo, F. , Díaz‐Almiron, M. , Vich‐Pérez, P. , San andrés‐Rebollo, F. J. , Vicente, I. , & Mostaza, J. M. (2022). HDL‐cholesterol concentration and risk of SARS‐CoV‐2 infection in people over 75 years of age: A cohort with half a million participants from the Community of Madrid. Clínica e Investigación en Arteriosclerosis (English Edition), 34(3), 113–119. 10.1016/j.artere.2022.05.002 PMC8654578 35125250

[jcp31424-bib-0050] Li, B. , Yang, J. , Zhao, F. , Zhi, L. , Wang, X. , Liu, L. , Bi, Z. , & Zhao, Y. (2020). Prevalence and impact of cardiovascular metabolic diseases on COVID‐19 in China. Clinical Research in Cardiology, 109(5), 531–538. 10.1007/s00392-020-01626-9 32161990 PMC7087935

[jcp31424-bib-0051] Li, M. , Dong, Y. , Wang, H. , Guo, W. , Zhou, H. , Zhang, Z. , Tian, C. , Du, K. , Zhu, R. , Wang, L. , Zhao, L. , Fan, H. , Luo, S. , & Hu, D. (2020). Cardiovascular disease potentially contributes to the progression and poor prognosis of COVID‐19. Nutrition, Metabolism, and Cardiovascular Diseases, 30(7), 1061–1067. 10.1016/j.numecd.2020.04.013 PMC7165120 32456948

[jcp31424-bib-0052] Li, S.‐S. , Gao, S. , Chen, Y. , Bao, H. , Li, Z.‐T. , Yao, Q.‐P. , Liu, J. T. , Wang, Y. , & Qi, Y. X. (2021). Platelet‐derived microvesicles induce calcium oscillations and promote VSMC migration via TRPV4. Theranostics, 11(5), 2410–2423. 33500733 10.7150/thno.47182 PMC7797689

[jcp31424-bib-0053] Li, X. , Zhu, W. , Fan, M. , Zhang, J. , Peng, Y. , Huang, F. , Wang, N. , He, L. , Zhang, L. , Holmdahl, R. , Meng, L. , & Lu, S. (2021). Dependence of SARS‐CoV‐2 infection on cholesterol‐rich lipid raft and endosomal acidification. Computational and Structural Biotechnology Journal, 19, 1933–1943. 33850607 10.1016/j.csbj.2021.04.001 PMC8028701

[jcp31424-bib-0054] Liu, M. , Wang, T. , Zhou, Y. , Zhao, Y. , Zhang, Y. , & Li, J. (2020). Potential role of ACE2 in coronavirus disease 2019 (COVID‐19) prevention and management. Journal of Translational Internal Medicine, 8(1), 9–19. 10.2478/jtim-2020-0003 32435607 PMC7227161

[jcp31424-bib-0055] Liu, M. H. , Lin, X. L. , & Xiao, L. L. (2024a). Excess phosphate promotes SARS‑CoV‑2 N protein‑induced NLRP3 inflammasome activation via the SCAP‑SREBP2 signaling pathway. Molecular Medicine Reports, 29(3). 10.3892/mmr.2024.13173 38275129

[jcp31424-bib-0056] Liu, M. H. , Lin, X. L. , & Xiao, L. L. (2024b). SARS‐CoV‐2 nucleocapsid protein promotes TMAO‐induced NLRP3 inflammasome activation by SCAP‐SREBP signaling pathway. Tissue and Cell, 86, 102276. 10.1016/j.tice.2023.102276 37979395

[jcp31424-bib-0057] Liu, Y. Z. , Li, Z. X. , Zhang, L. L. , Wang, D. , & Liu, Y. P. (2022). Phenotypic plasticity of vascular smooth muscle cells in vascular calcification: Role of mitochondria. Frontiers in Cardiovascular Medicine, 9, 972836. 10.3389/fcvm.2022.972836 36312244 PMC9597684

[jcp31424-bib-0058] Luo, S. , Qiu, X. M. , Zeng, X. J. , Zhang, D. Y. , Wan, B. , Li, X. , Tian, R. H. , Wang, J. T. , Wang, M. Y. , Zhu, J. , Zhang, C. , Yang, R. , Chen, F. , Liang, Y. , Fan, B. , Jiang, H. J. , Wang, X. M. , Chen, W. , Xu, K. , … Lu, G. M. (2022). Coronary artery calcification and risk of mortality and adverse outcomes in patients with COVID‐19: A Chinese multicenter retrospective cohort study. Chinese Journal of Academic Radiology, 5(1), 20–28. 10.1007/s42058-021-00072-4 34222797 PMC8237549

[jcp31424-bib-0059] Mao, S. , Ren, J. , Xu, Y. , Lin, J. , Pan, C. , Meng, Y. , & Xu, N. (2022). Studies in the antiviral molecular mechanisms of 25‐hydroxycholesterol: Disturbing cholesterol homeostasis and post‐translational modification of proteins. European Journal of Pharmacology, 926, 175033. 35598845 10.1016/j.ejphar.2022.175033 PMC9119167

[jcp31424-bib-0060] Marcello, A. , Civra, A. , Milan Bonotto, R. , Nascimento Alves, L. , Rajasekharan, S. , Giacobone, C. , Caccia, C. , Cavalli, R. , Adami, M. , Brambilla, P. , Lembo, D. , Poli, G. , & Leoni, V. (2020). The cholesterol metabolite 27‐hydroxycholesterol inhibits SARS‐CoV‐2 and is markedly decreased in COVID‐19 patients. Redox Biology, 36, 101682. 32810737 10.1016/j.redox.2020.101682 PMC7416714

[jcp31424-bib-0061] Martínez‐Salazar, B. , Holwerda, M. , Stüdle, C. , Piragyte, I. , Mercader, N. , Engelhardt, B. , Rieben, R. , & Döring, Y. (2022). COVID‐19 and the vasculature: Current aspects and long‐term consequences. Frontiers in Cell and Developmental Biology, 10:824851. 10.3389/fcell.2022.824851 35242762 PMC8887620

[jcp31424-bib-0062] Mehra, M. R. , & Ruschitzka, F. (2020). COVID‐19 illness and heart failure. JACC Heart Failure, 8(6), 512–514. 10.1016/j.jchf.2020.03.004 32360242 PMC7151428

[jcp31424-bib-0063] Meyer, H. J. , Gottschling, S. , Borggrefe, J. , & Surov, A. (2023). CT coronary artery calcification score as a prognostic marker in COVID‐19. Journal of Thoracic Disease, 15(10), 5559–5565. 10.21037/jtd-23-728 37969270 PMC10636427

[jcp31424-bib-0064] Mokas, S. , Larivière, R. , Lamalice, L. , Gobeil, S. , Cornfield, D. N. , Agharazii, M. , & Richard, D. E. (2016). Hypoxia‐inducible factor‐1 plays a role in phosphate‐induced vascular smooth muscle cell calcification. Kidney International, 90(3), 598–609. 27470678 10.1016/j.kint.2016.05.020

[jcp31424-bib-0065] Moser, B. , Poetsch, F. , Estepa, M. , Luong, T. T. D. , Pieske, B. , Lang, F. , Alesutan, I. , & Voelkl, J. (2021). Increased β‐adrenergic stimulation augments vascular smooth muscle cell calcification via PKA/CREB signalling. Pflügers Archiv‐European Journal of Physiology, 473, 1899–1910. 34564739 10.1007/s00424-021-02621-3 PMC8599266

[jcp31424-bib-0066] Naeem, A. , Tabassum, S. , Gill, S. , Khan, M. Z. , Mumtaz, N. , Qaiser, Q. , Karamat, M. , Arif, M. , Naeem, F. , Afifi, A. , Basit, J. , & Nashwan, A. J. (2023). COVID‐19 and cardiovascular diseases: A literature review from pathogenesis to diagnosis. Cureus, 15(3), e35658. 10.7759/cureus.35658 37009373 PMC10065369

[jcp31424-bib-0067] Nishiga, M. , Wang, D. W. , Han, Y. , Lewis, D. B. , & Wu, J. C. (2020). COVID‐19 and cardiovascular disease: From basic mechanisms to clinical perspectives. Nature Reviews Cardiology, 17(9), 543–558. 10.1038/s41569-020-0413-9 32690910 PMC7370876

[jcp31424-bib-0068] Opdebeeck, B. , Orriss, I. R. , Neven, E. , D'Haese, P. C. , & Verhulst, A. (2020). Extracellular nucleotides regulate arterial calcification by activating both independent and dependent purinergic receptor signaling pathways. International Journal of Molecular Sciences, 21(20), 7636. 33076470 10.3390/ijms21207636 PMC7589647

[jcp31424-bib-0069] Pai, A. , Leaf, E. M. , El‐Abbadi, M. , & Giachelli, C. M. (2011). Elastin degradation and vascular smooth muscle cell phenotype change precede cell loss and arterial medial calcification in a uremic mouse model of chronic kidney disease. The American Journal of Pathology, 178(2), 764–773. 10.1016/j.ajpath.2010.10.006 21281809 PMC3069837

[jcp31424-bib-0070] Palacios‐Rápalo, S. N. , De jesús‐González, L. A. , Cordero‐Rivera, C. D. , Farfan‐Morales, C. N. , Osuna‐Ramos, J. F. , Martínez‐Mier, G. , Quistián‐Galván, J. , Muñoz‐Pérez, A. , Bernal‐Dolores, V. , del Ángel, R. M. , & Reyes‐Ruiz, J. M. (2021). Cholesterol‐rich lipid rafts as platforms for SARS‐CoV‐2 entry. Frontiers in Immunology, 12:796855. 34975904 10.3389/fimmu.2021.796855 PMC8719300

[jcp31424-bib-0071] Parums, D. V. (2022). Editorial: Cardiovascular complications at one year after SARS‐CoV‐2 infection are independent of underlying cardiovascular risk factors or severity of COVID‐19. Medical Science Monitor, 28:e937048. 10.12659/msm.937048 35490309 PMC9069970

[jcp31424-bib-0072] Patel, J. J. , Zhu, D. , Opdebeeck, B. , D'Haese, P. , Millán, J. L. , Bourne, L. E. , Wheeler‐Jones, C. P. D. , Arnett, T. R. , MacRae, V. E. , & Orriss, I. R. (2018). Inhibition of arterial medial calcification and bone mineralization by extracellular nucleotides: The same functional effect mediated by different cellular mechanisms. Journal of Cellular Physiology, 233(4), 3230–3243. 10.1002/jcp.26166 28976001 PMC5792173

[jcp31424-bib-0073] Pelisek, J. , Reutersberg, B. , Greber, U. F. , & Zimmermann, A. (2022). Vascular dysfunction in COVID‐19 patients: Update on SARS‐CoV‐2 infection of endothelial cells and the role of long non‐coding RNAs. Clinical Science, 136(21), 1571–1590. 10.1042/cs20220235 36367091 PMC9652506

[jcp31424-bib-0074] Pizzato, M. , Baraldi, C. , Boscato Sopetto, G. , Finozzi, D. , Gentile, C. , Gentile, M. D. , Marconi, R. , Paladino, D. , Raoss, A. , Riedmiller, I. , Ur Rehman, H. , Santini, A. , Succetti, V. , & Volpini, L. (2022). SARS‐CoV‐2 and the host cell: A tale of interactions. Frontiers in Virology, 1.

[jcp31424-bib-0075] Possari, R. Y. , Andrade‐Gomes, H. J. , Mello, V. C. , Galdeano, E. A. , Aguiar‐Filho, L. F. , Bittencourt, M. S. , Ponte, E. V. , Bertoche, L. R. , Caio, L. , Rodrigues, J. D. , Alcantara, F. B. , Freitas, M. , Sarinho, J. , Cervigne, N. K. , Rodrigues, W. M. , & Aprahamian, I. (2021). Association of coronary calcification with prognosis of Covid‐19 patients without known heart disease. Brazilian Journal of Medical and Biological Research = Revista Brasileira de Pesquisas Medicas e Biologicas, 54(12), e11681. 10.1590/1414-431X2021e11681 34878066 PMC8647900

[jcp31424-bib-0076] Poznyak, A. V. , Bezsonov, E. E. , Eid, A. H. , Popkova, T. V. , Nedosugova, L. V. , Starodubova, A. V. , & Orekhov, A. N. (2021). ACE2 is an adjacent element of atherosclerosis and COVID‐19 pathogenesis. International Journal of Molecular Sciences, 22(9), 4691. 10.3390/ijms22094691 33946649 PMC8124184

[jcp31424-bib-0077] Prado, K. B. , Shugg, S. , & Backstrand, J. R. (2011). Low‐density lipoprotein particle number predicts coronary artery calcification in asymptomatic adults at intermediate risk of cardiovascular disease. Journal of Clinical Lipidology, 5(5), 408–413. 21981843 10.1016/j.jacl.2011.07.001

[jcp31424-bib-0078] Roman‐Garcia, P. , Barrio‐Vazquez, S. , Fernandez‐Martin, J. L. , Ruiz‐Torres, M. P. , & Cannata‐Andia, J. B. (2011). Natural antioxidants and vascular calcification: A possible benefit. Journal of Nephrology, 24(6), 669–672. 21928237 10.5301/jn.5000029

[jcp31424-bib-0079] da Rosa Mesquita, R. , Francelino Silva Junior, L. C. , Santos Santana, F. M. , Farias de Oliveira, T. , Campos Alcântara, R. , Monteiro Arnozo, G. , Rodrigues da Silva Filho, E. , Galdino Dos Santos, A. G. , Oliveira da Cunha, E. J. , Salgueiro de Aquino, S. H. , & Freire de Souza, C. D. (2021). Clinical manifestations of COVID‐19 in the general population: Systematic review. Wiener Klinische Wochenschrift, 133(7–8), 377–382. 10.1007/s00508-020-01760-4 33242148 PMC7689634

[jcp31424-bib-0080] Salabei, J. K. , Asnake, Z. T. , Ismail, Z. H. , Charles, K. , Stanger, G. T. , Abdullahi, A. H. , Abraham, A. T. , & Okonoboh, P. (2022). COVID‐19 and the cardiovascular system: An update. The American Journal of the Medical Sciences, 364(2), 139–147. 10.1016/j.amjms.2022.01.022 35151635 PMC8830924

[jcp31424-bib-0081] Sanders, D. W. , Jumper, C. C. , Ackerman, P. J. , Bracha, D. , Donlic, A. , Kim, H. , Kenney, D. , Castello‐Serrano, I. , Suzuki, S. , Tamura, T. , Tavares, A. H. , Saeed, M. , Holehouse, A. S. , Ploss, A. , Levental, I. , Douam, F. , Padera, R. F. , Levy, B. D. , & Brangwynne, C. P. (2021). SARS‐CoV‐2 requires cholesterol for viral entry and pathological syncytia formation. eLife, 10. 10.7554/eLife.65962 PMC8104966 33890572

[jcp31424-bib-0082] Sanyour, H. J. , Li, N. , Rickel, A. P. , Torres, H. M. , Anderson, R. H. , Miles, M. R. , Childs, J. D. , Francis, K. R. , Tao, J. , & Hong, Z. (2020). Statin‐mediated cholesterol depletion exerts coordinated effects on the alterations in rat vascular smooth muscle cell biomechanics and migration. The Journal of Physiology, 598(8), 1505–1522. 10.1113/JP279528 32083311 PMC7394245

[jcp31424-bib-0083] Schiavone, M. , Gobbi, C. , Biondi‐Zoccai, G. , D'ascenzo, F. , Palazzuoli, A. , Gasperetti, A. , Mitacchione, G. , Viecca, M. , Galli, M. , Fedele, F. , Mancone, M. , & Forleo, G. B. (2020). Acute coronary syndromes and Covid‐19: Exploring the uncertainties. Journal of Clinical Medicine, 9(6), 1683. 10.3390/jcm9061683 32498230 PMC7356537

[jcp31424-bib-0084] Schiffl, H. , & Lang, S. M. (2023). Long‐term interplay between COVID‐19 and chronic kidney disease. International Urology and Nephrology, 55(8), 1977–1984. 10.1007/s11255-023-03528-x 36828919 PMC9955527

[jcp31424-bib-0085] SeyedAlinaghi, S. , Afsahi, A. M. , MohsseniPour, M. , Behnezhad, F. , Salehi, M. A. , Barzegary, A. , Mirzapour, P. , Mehraeen, E. , & Dadras, O. (2021). Late complications of COVID‐19; a systematic review of current evidence. Archives of Academic Emergency Medicine, 9(1), 14. 10.22037/aaem.v9i1.1058 PMC7927752 33681819

[jcp31424-bib-0086] Shabestari, A. , Mahdavi, A. , Abrishami, A. , Alahyari, S. , & Molla, M. (2022). Coronary artery calcification: Effects on severity and survival in patients with COVID‐19. Journal of Research in Medical Sciences, 27, 89. 10.4103/jrms.jrms_584_21 36685025 PMC9854912

[jcp31424-bib-0087] Slipczuk, L. , Castagna, F. , Schonberger, A. , Novogrodsky, E. , Sekerak, R. , Dey, D. , Jorde, U. P. , Levsky, J. M. , & Garcia, M. J. (2021). Coronary artery calcification and epicardial adipose tissue as independent predictors of mortality in COVID‐19. The International Journal of Cardiovascular Imaging, 37(10), 3093–3100. 10.1007/s10554-021-02276-2 33978937 PMC8113796

[jcp31424-bib-0088] Soumya, R. S. , Unni, T. G. , & Raghu, K. G. (2021). Impact of COVID‐19 on the cardiovascular system: A review of available reports. Cardiovascular Drugs and Therapy, 35(3), 411–425. 10.1007/s10557-020-07073-y 32926272 PMC7487338

[jcp31424-bib-0089] Straus, M. R. , Bidon, M. K. , Tang, T. , Jaimes, J. A. , Whittaker, G. R. , & Daniel, S. (2021). Inhibitors of L‐type calcium channels show therapeutic potential for treating SARS‐CoV‐2 infections by preventing virus entry and spread. ACS Infectious Diseases, 7(10), 2807–2815. 10.1021/acsinfecdis.1c00023 34498840

[jcp31424-bib-0090] Su, X. , Cheng, Y. , Zhang, G. , & Wang, B. (2021). Chemerin in inflammatory diseases. Clinica Chimica Acta, 517, 41–47. 10.1016/j.cca.2021.02.010 33631197

[jcp31424-bib-0091] Sultan, F. , Ahuja, K. , & Motiani, R. K. (2022). Potential of targeting host cell calcium dynamics to curtail SARS‐CoV‐2 infection and COVID‐19 pathogenesis. Cell Calcium, 106, 102637. 35986958 10.1016/j.ceca.2022.102637 PMC9367204

[jcp31424-bib-0092] Sun, J.‐X. , Zhang, C. , Cheng, Z.‐B. , Tang, M.‐Y. , Liu, Y.‐Z. , Jiang, J.‐F. , Xiao, X. , & Huang, L. (2021). Chemerin in atherosclerosis. Clinica Chimica Acta, 520, 8–15. 10.1016/j.cca.2021.05.015 34022243

[jcp31424-bib-0093] Suzuki, Y. J. , Nikolaienko, S. I. , Dibrova, V. A. , Dibrova, Y. V. , Vasylyk, V. M. , Novikov, M. Y. , Shults, N. V. , & Gychka, S. G. (2021). SARS‐CoV‐2 spike protein‐mediated cell signaling in lung vascular cells. Vascular Pharmacology, 137:106823. 10.1016/j.vph.2020.106823 33232769 PMC7680014

[jcp31424-bib-0094] Sykes, R. A. , Neves, K. B. , Alves‐Lopes, R. , Caputo, I. , Fallon, K. , Jamieson, N. B. , Kamdar, A. , Legrini, A. , Leslie, H. , McIntosh, A. , McConnachie, A. , Morrow, A. , McFarlane, R. W. , Mangion, K. , McAbney, J. , Montezano, A. C. , Touyz, R. M. , Wood, C. , & Berry, C. (2023). Vascular mechanisms of post‐COVID‐19 conditions: Rho‐kinase is a novel target for therapy. European Heart Journal. Cardiovascular Pharmacotherapy, 9(4), 371–386. 10.1093/ehjcvp/pvad025 37019821 PMC10236521

[jcp31424-bib-0095] Tajbakhsh, A. , Gheibi Hayat, S. M. , Taghizadeh, H. , Akbari, A. , Inabadi, M. , Savardashtaki, A. , Johnston, T. P. , & Sahebkar, A. (2021). COVID‐19 and cardiac injury: Clinical manifestations, biomarkers, mechanisms, diagnosis, treatment, and follow up. Expert Review of Anti‐Infective Therapy, 19(3), 345–357. 10.1080/14787210.2020.1822737 32921216

[jcp31424-bib-0096] Tang, H. Y. , Chen, A. Q. , Zhang, H. , Gao, X. F. , Kong, X. Q. , & Zhang, J. J. (2022). Vascular smooth muscle cells phenotypic switching in cardiovascular diseases. Cells, 11(24). 10.3390/cells11244060 PMC9777337 36552822

[jcp31424-bib-0097] Tang, T. , Bidon, M. , Jaimes, J. A. , Whittaker, G. R. , & Daniel, S. (2020). Coronavirus membrane fusion mechanism offers a potential target for antiviral development. Antiviral Research, 178, 104792. 32272173 10.1016/j.antiviral.2020.104792 PMC7194977

[jcp31424-bib-0098] Toft‐Bertelsen, T. L. , Yarishkin, O. , Redmon, S. , Phuong, T. T. T. , Križaj, D. , & MacAulay, N. (2019). Volume sensing in the transient receptor potential vanilloid 4 ion channel is cell type‐specific and mediated by an N‐terminal volume‐sensing domain. Journal of Biological Chemistry, 294(48), 18421–18434. 31619514 10.1074/jbc.RA119.011187 PMC6885641

[jcp31424-bib-0099] Tsai, Y.‐T. , Yeh, H.‐Y. , Chao, C.‐T. , & Chiang, C.‐K. (2021). Superoxide dismutase 2 (SOD2) in vascular calcification: A focus on vascular smooth muscle cells, calcification pathogenesis, and therapeutic strategies. Oxidative Medicine and Cellular Longevity, 2021, 1–9. 10.1155/2021/6675548 PMC7935587 33728027

[jcp31424-bib-0100] Vengrenyuk, Y. , Nishi, H. , Long, X. , Ouimet, M. , Savji, N. , Martinez, F. O. , Cassella, C. P. , Moore, K. J. , Ramsey, S. A. , Miano, J. M. , & Fisher, E. A. (2015). Cholesterol loading reprograms the microRNA‐143/145–myocardin axis to convert aortic smooth muscle cells to a dysfunctional macrophage‐like phenotype. Arteriosclerosis, Thrombosis, and Vascular Biology, 35(3), 535–546. 25573853 10.1161/ATVBAHA.114.304029 PMC4344402

[jcp31424-bib-0101] Wang, S. , Li, W. , Hui, H. , Tiwari, S. K. , Zhang, Q. , Croker, B. A. , Rawlings, S. , Smith, D. , Carlin, A. F. , & Rana, T. M. (2020). Cholesterol 25‐Hydroxylase inhibits SARS‐CoV‐2 and other coronaviruses by depleting membrane cholesterol. The EMBO Journal, 39(21), e106057. 10.15252/embj.2020106057 32944968 PMC7537045

[jcp31424-bib-0102] Wehbe, Z. , Hammoud, S. H. , Yassine, H. M. , Fardoun, M. , El‐Yazbi, A. F. , & Eid, A. H. (2021). Molecular and biological mechanisms underlying gender differences in COVID‐19 severity and mortality. Frontiers in Immunology, 12:659339. 10.3389/fimmu.2021.659339 34025658 PMC8138433

[jcp31424-bib-0103] Wehbe, Z. , Wehbe, M. , Iratni, R. , Pintus, G. , Zaraket, H. , Yassine, H. M. , & Eid, A. H. (2021). Repurposing ivermectin for COVID‐19: Molecular aspects and therapeutic possibilities. Frontiers in Immunology, 12:663586. 10.3389/fimmu.2021.663586 33859652 PMC8043070

[jcp31424-bib-0104] Wei, H.‐F. , Anchipolovsky, S. , Vera, R. , Liang, G. , & Chuang, D.‐M. (2022). Potential mechanisms underlying lithium treatment for Alzheimer's disease and COVID‐19. European Review for Medical and Pharmacological Sciences, 26(6), 2201–2214. 35363371 10.26355/eurrev_202203_28369 PMC9173589

[jcp31424-bib-0105] Xu, S. W. , Ilyas, I. , & Weng, J. P. (2023). Endothelial dysfunction in COVID‐19: An overview of evidence, biomarkers, mechanisms and potential therapies. Acta Pharmacologica Sinica, 44(4), 695–709. 10.1038/s41401-022-00998-0 36253560 PMC9574180

[jcp31424-bib-0106] Younis, N. K. , Zareef, R. O. , Al Hassan, S. N. , Bitar, F. , Eid, A. H. , & Arabi, M. (2020). Hydroxychloroquine in COVID‐19 patients: Pros and cons. Frontiers in Pharmacology, 11:597985. 10.3389/fphar.2020.597985 33364965 PMC7751757

[jcp31424-bib-0107] Younis, N. K. , Zareef, R. O. , Fakhri, G. , Bitar, F. , Eid, A. H. , & Arabi, M. (2021). COVID‐19: Potential therapeutics for pediatric patients. Pharmacological Reports, 73(6), 1520–1538. 10.1007/s43440-021-00316-1 34458951 PMC8403523

[jcp31424-bib-0108] Zaid, M. , Miura, K. , Fujiyoshi, A. , Abbott, R. D. , Hisamatsu, T. , Kadota, A. , Arima, H. , Kadowaki, S. , Torii, S. , Miyagawa, N. , Suzuki, S. , Takashima, N. , Ohkubo, T. , Sekikawa, A. , Maegawa, H. , Horie, M. , Nakamura, Y. , Okamura, T. , Ueshima, H. , … Ito, T. (2016). Associations of serum LDL particle concentration with carotid intima‐media thickness and coronary artery calcification. Journal of Clinical Lipidology, 10(5), 1195–1202.e1. e1191. 10.1016/j.jacl.2015.12.027 27678437 PMC5047009

[jcp31424-bib-0109] Zang, R. , Case, J. B. , Yutuc, E. , Ma, X. , Shen, S. , Gomez Castro, M. F. , Liu, Z. , Zeng, Q. , Zhao, H. , Son, J. , Rothlauf, P. W. , Kreutzberger, A. J. B. , Hou, G. , Zhang, H. , Bose, S. , Wang, X. , Vahey, M. D. , Mani, K. , Griffiths, W. J. , … Ding, S. (2020). Cholesterol 25‐hydroxylase suppresses SARS‐CoV‐2 replication by blocking membrane fusion. Proceedings of the National Academy of Sciences United States of America, 117(50), 32105–32113. 10.1073/pnas.2012197117 PMC7749331 33239446

[jcp31424-bib-0110] Zareef, R. O. , Younis, N. K. , Bitar, F. , Eid, A. H. , & Arabi, M. (2020). COVID‐19 in pediatric patients: A focus on CHD patients. Frontiers in Cardiovascular Medicine, 7:612460. 10.3389/fcvm.2020.612460 33330675 PMC7728667

[jcp31424-bib-0111] Zhang, L. , Li, M. , Wang, Z. , Sun, P. , Wei, S. , Zhang, C. , Wu, H. , & Bai, H. (2021). Cardiovascular risk after SARS‐CoV‐2 infection is mediated by IL18/IL18R1/HIF‐1 signaling pathway axis. Frontiers in Immunology, 12:780804. 10.3389/fimmu.2021.780804 35069552 PMC8766743

[jcp31424-bib-0112] Zhang, M.‐J. , Zhou, Y. , Chen, L. , Wang, Y.‐Q. , Wang, X. , Pi, Y. , Gao, C. Y. , Li, J. C. , & Zhang, L. L. (2016). An overview of potential molecular mechanisms involved in VSMC phenotypic modulation. Histochemistry and Cell Biology, 145, 119–130. 26708152 10.1007/s00418-015-1386-3

[jcp31424-bib-0113] Zhang, Q. , Chen, C. Z. , Swaroop, M. , Xu, M. , Wang, L. , Lee, J. , Pradhan, M. , Shen, M. , Luo, Z. , Xu, Y. , Huang, W. , Zheng, W. , & Ye, Y. (2020). Targeting heparan sulfate proteoglycan‐assisted endocytosis as a COVID‐19 therapeutic option. bioRxiv . 10.1101/2020.07.14.202549

[jcp31424-bib-0114] Zhou, F. , Yu, T. , Du, R. , Fan, G. , Liu, Y. , Liu, Z. , Xiang, J. , Wang, Y. , Song, B. , Gu, X. , Guan, L. , Wei, Y. , Li, H. , Wu, X. , Xu, J. , Tu, S. , Zhang, Y. , Chen, H. , & Cao, B. (2020). Clinical course and risk factors for mortality of adult inpatients with COVID‐19 in Wuhan, China: A retrospective cohort study. The Lancet, 395(10229), 1054–1062. 10.1016/S0140-6736(20)30566-3 PMC7270627 32171076

[jcp31424-bib-0115] Zimna, A. , & Kurpisz, M. (2015). Hypoxia‐inducible factor‐1 in physiological and pathophysiological angiogenesis: Applications and therapies. BioMed Research International, 2015, 1–13. 10.1155/2015/549412 PMC4471260 26146622

[jcp31424-bib-0116] Zipeto, D. , Palmeira, J. F. , Argañaraz, G. A. , & Argañaraz, E. R. (2020). ACE2/ADAM17/TMPRSS2 interplay may be the main risk factor for COVID‐19. Frontiers in Immunology, 11, 576745. 33117379 10.3389/fimmu.2020.576745 PMC7575774

